# Salvianolic acid A inhibits PRRSV replication via binding to Keap1 to activate the MKRN1–Nrf2–NQO1 pathway

**DOI:** 10.1186/s13567-025-01614-9

**Published:** 2025-09-25

**Authors:** Hong Duan, Yaci Zhang, Aijuan Shen, Jiahui Ren, Fengxia Zhang, Yunshuo Lu, Xuedan Wei, Chaoyu Yang, Jiexi Gong, Xin Wang, Yongkun Du, Qiming Pei, Angke Zhang

**Affiliations:** 1 College of Veterinary Medicine, Henan Agricultural University, Zhengzhou, China; 2 International Joint Research Center of National Animal Immunology, College of Veterinary Medicine, Henan Agricultural University, Zhengzhou, China; 3 Ministry of Education Key Laboratory for Animal Pathogens and Biosafety, College of Veterinary Medicine, Henan Agricultural University, Zhengzhou, China; 4 Longhu Laboratory of Advanced Immunology, Zhengzhou, China

**Keywords:** Porcine reproductive and respiratory syndrome virus, salvianolic acid A, lipopolysaccharide, antiviral activity, anti-inflammatory activity

## Abstract

Porcine reproductive and respiratory syndrome (PRRS) has caused significant economic losses to the global pig industry, and there are currently no safe, effective, or commercially available vaccines. Traditional Chinese medicine may serve as a beneficial supplement to vaccines and provide a feasible solution for preventing and controlling PRRS. The present study explored the effects and molecular mechanisms of the traditional Chinese medicine *Salviae miltiorrhizae* Bunge extract salvianolic acid A (SalA) on porcine reproductive and respiratory syndrome virus (PRRSV) replication. SalA effectively inhibited PRRSV replication in vitro and in vivo without affecting the adsorption, entry, or release stages of the viral replication cycle. SalA directly binds to the Thr560 site of Kelch-like ECH-associated protein 1 (Keap1) via a hydrogen bond, promoting the recruitment of Keap1 to a newly identified E3 ubiquitin ligase, makorin RING finger protein 1 (MKRN1). This led to K48-linked ubiquitination of Keap1 at the K615 amino acid residue, subsequent proteasomal degradation, and eventual activation of the nuclear factor E2-related factor 2 (Nrf2)-NADPH quinone oxidoreductase 1 (NQO1) pathway. Knockdown of MKRN1 blocked SalA-induced Keap1 ubiquitination, degradation, and activation of the Nrf2-NQO1 pathway. Further viral infection experiments revealed that SalA inhibited PRRSV replication by activating the MKRN1-Nrf2-NQO1 pathway. In addition to its anti-PRRSV activity, SalA effectively inhibited PRRSV- and lipopolysaccharide (LPS)-induced expression of inflammatory cytokines, activation of inflammatory pathways and inflammasomes, and inhibition of cellular pyroptosis by activating the Nrf2 pathway. These results suggest that SalA can inhibit PRRSV replication and alleviate the inflammatory response during PRRS and secondary bacterial infections, providing a novel candidate strategy for PRRS treatment.

**Graphical Abstract:**

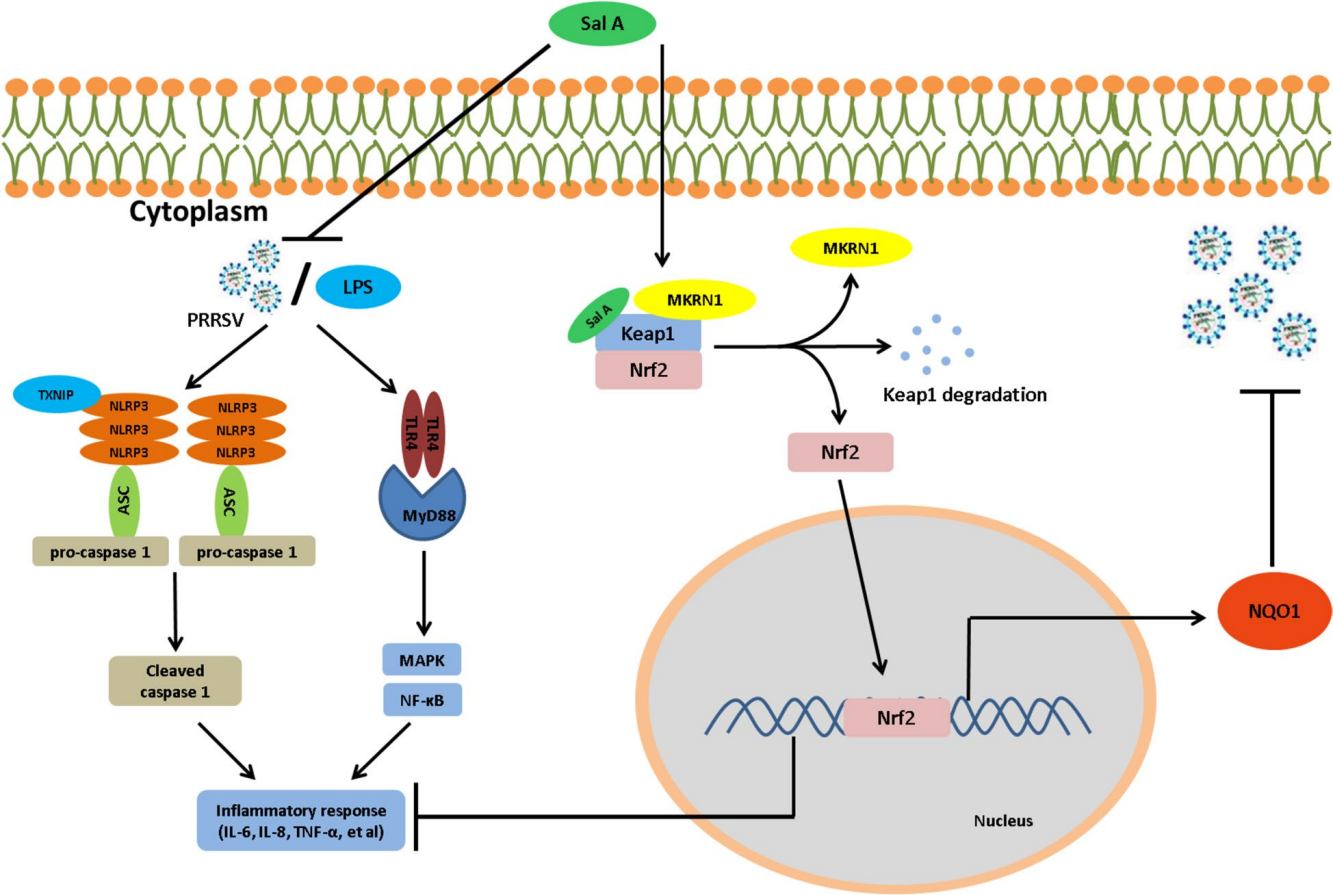

**Supplementary Information:**

The online version contains supplementary material available at 10.1186/s13567-025-01614-9.

## Introduction

Porcine reproductive and respiratory syndrome (PRRS) is an acute and highly transmissible disease caused by porcine reproductive and respiratory syndrome virus (PRRSV) [1]. PRRS has caused enormous economic losses to the global swine industry. In China, it has long been one of the most harmful diseases on pig farms since the large-scale outbreak and epidemic of highly pathogenic PRRSV (HP-PRRSV) in 2006 [2–4]. The typical clinical symptoms of PRRS include reproductive disorders in sows and acute respiratory diseases in pigs of all ages [5]. To date, vaccination remains one of the key measures for the prevention and control of PRRSV on most pig farms. Two types of vaccines are used for PRRSV prevention worldwide: live-attenuated vaccines and inactivated vaccines. Nevertheless, live attenuated vaccines only protect against homologous strains and pose many biosafety concerns due to the risk of recombination between wild viruses and vaccine strains or the inherent mutational tendency of PRRSV, whereas inactivated vaccines fail to induce sufficiently strong neutralizing antibodies [6]. Therefore, alternative strategies to control PRRSV and reduce economic losses are urgently needed.

PRRSV is an enveloped, single-stranded positive-sense RNA virus belonging to the genus *Porartevirus*, family *Arteriviridae*, and order *Nidovirales* and can be divided into type 1 and type 2 [7, 8]. PRRSV infection is always accompanied by high levels of proinflammatory cytokines in vivo, such as interleukin (IL)-1β, IL-6, IL-8, and tumour necrosis factor alpha (TNF-α) [9–14], which play pivotal roles in the tissue damage induced by systemic inflammation. High-mobility group box 1 protein (HMGB1) is a member of the damage-associated molecular pattern (DAMP) family. It induces NLRP3 inflammasome activation, which leads to increased inflammation and aggravated tissue damage [15]. Pigs infected with HP-PRRSV are often complicated by secondary bacterial infections, which significantly exacerbate severe clinical symptoms and pathological injury, such as high fever and acute lung injury. Gram-negative bacteria, such as *Escherichia coli*, *Haemophilus parasuis*, *Vibrio cholerae*, *Bacillus bronchisepticum*, and *Pasteurella multocida*, frequently cause secondary infections in HP-PRRSV-infected pigs and can release high concentrations of lipopolysaccharide (LPS) in the lungs [16–18]. The presence of LPS further promotes the secretion of inflammatory cytokines and exacerbates lung injury and respiratory syndrome induced by PRRSV [19].

The Toll-like receptor (TLR) family is a major pattern recognition receptor (PRR) family capable of recognizing various pathogen-associated molecular patterns (PAMPs) present in bacteria or viruses, thereby activating specific signalling pathways that evoke the transcription and expression of inflammatory and/or anti-inflammatory cytokines. Myeloid differentiation factor 88 (MyD88) interacts with TLR4 to activate nuclear factor kappa B (NF-κB), promoting the release of inflammatory mediators and the initiation of the immune response [20]. The principal mitogen-activated protein kinases (MAPKs) implicated in the above process include extracellular signal-regulated kinase (ERK), c-Jun N-terminal kinase (JNK), and P38 MAPK. All of these factors are regulated in response to inflammatory cytokines [21] and are closely associated with PRRSV-induced inflammatory responses [22, 23].

The nucleotide-binding oligomerization domain (NOD)-like receptor protein 3 (NLRP3) inflammasome is strongly associated with inflammation and pyroptosis [24]. Upon stimulation, initiating signals activate the transcription and posttranslational modifications of NLRP3, pro-IL-1β, and pro-IL-18 [25, 26]. Subsequently, the ASC (apoptosis-associated speck-like protein containing a CARD) adaptor, which possesses a caspase recruitment domain (CARD), connects the sensor to the downstream effector caspase-1, thereby facilitating the production of mature IL-1β and IL-18. Furthermore, Txnip acts as an upstream regulator of NLRP3 and activates the NLRP3 inflammasome, which in turn activates caspase-1 and IL-18, leading to cleavage of the amino-terminal (GSDMD-N) and carboxyl-terminal (GSDMD-C) domains of GSDMD, ultimately triggering cellular pyroptosis [27]. Studies have shown that PRRSV infection activates NLRP3-dependent pyroptosis in porcine alveolar macrophages (PAMs) [9].

Compared with chemically synthesized pharmaceuticals, traditional Chinese medicine has many natural advantages, such as a wide range of sources, easy availability, low cost, minimal side effects, minimal residual compounds, and a lack of drug resistance. Salvianolic acid A (SalA) is a minor phenolic carboxylic acid extracted from *Salviae miltiorrhizae* Bunge (Danshen in Chinese). As one of the major active components of this plant, SalA has multiple pharmacological activities, such as antioxidative effects [28, 29] and anti-inflammatory effects [30, 31]. In addition, SalA inhibits infection by the pseudorabies virus (PRV), a viral pathogen of animal hosts, by directly inactivating viral infectivity [32]. SalA inhibits SARS-CoV-2 spike pseudovirus entry into ACE2^h^ cells by binding to the viral spike protein’s receptor-binding domain (RBD) and the ACE2 protein [33]. However, the role of SalA in PRRSV infection and replication has not been studied to date, and there are no commercial anti-PRRSV drugs available for the pig breeding industry.

The present study explored the effects of SalA on PRRSV replication, PRRSV-induced inflammation and pyroptosis, as well as the underlying molecular mechanism. SalA inhibited PRRSV replication by activating the MKRN1-Nrf2-NQO1 pathway in vitro. Further analysis revealed that SalA suppressed the PRRSV-induced inflammatory response, inflammasome activation, and pyroptosis by inhibiting the TLR4/MyD88-NF-κB and TLR4/MyD88-MAPK and NLRP3-caspase-1/GSDMD signalling pathways and that Nrf2 played a central role in mediating the activity of SalA.

## Materials and methods

### Reagents, antibodies and viruses

Salvianolic acid A (HY-N0318), the proteasome pathway inhibitor MG-132 (HY-13259), the irreversible caspase-1 inhibitor Z-VAD-FMK (HY-16658B), the autophagy inhibitor 3-methyladenine (3-MA, HY-19312), the protein synthesis inhibitor cycloheximide (CHX, HY-12320), and the protein-degrading enzyme pronase were all purchased from MedChemExpress (Shanghai, China). NP-40 lysis buffer (P0013F) and the protease inhibitor phenylmethanesulfonyl fluoride (PMSF, ST2573) were obtained from Beyotime Biotechnology (Shanghai, China). Dimethyl sulfoxide (DMSO, D8371), lipopolysaccharide (LPS, L8880), and puromycin (P8230) were obtained from Solarbio (Beijing, China).

Antibodies against Myc-tag (AF2865), HA-tag (AF2858), p-ERK (AF5818), p-P65 (AF5875), P65 (AF1234), p-IκBα (AF5851), IκBα (AF5204), JNK (AF1048), P38 (AF7668), p-P38 (AF5887), TLR4 (AF8187), MyD88 (AF7524), Txnip (AF8277), NLRP3 (AF2155), and p-JNK (K006234) were all purchased from Beyotime Biotechnology. Antibodies against α-tubulin (11224-1-AP), Nrf2 (16396-1-AP), Keap1 (10503-2-AP), IL-1β (16,806–1-AP), ASC (10500-1-AP), and caspase-1 (22915-1-AP) were purchased from Proteintech Group (Wuhan, China). The antibody against GSDMD (ab210070) was obtained from Abcam (Cambridge, UK). Additionally, antibodies against NQO1 (49364-1) and HRP-conjugated goat anti-mouse IgG (H&L)/rabbit IgG (H&L) were obtained from Signalway Antibody (Texas, USA). Antibodies against PRRSV nucleocapsid (N) and nonstructural protein 4 (Nsp4) were prepared and preserved in our laboratory.

The HA-Ubiquitin, HA-Ubiquitin-K48, HA-Ubiquitin-K63, V5-Ubiquitin-K48R, V5-Ubiquitin-K63R, and GFP-Nrf2 plasmids were constructed in our laboratory. The recombinant plasmids pTrip-NQO1-Myc and pTrip-Keap1-K108R-Myc, -K298R-Myc, and -K615R-Myc were constructed in our laboratory. The plasmids pCMV-MKRN1-3 × Flag, -MKRN3-3 × Flag, and -IRF2BP1-3 × Flag were preserved in our laboratory.

A specific set of small interfering RNAs (siRNAs) targeting monkey *Nrf2* (sense: 5’-AAGAGUAUGAGCUGGAAAAAC -3’; antisense: 5’-GUUUUUCCAGCUCAUACUCUU-3’), *Nqo1* (sense: 5’-GAUUCUUAAUGAAAAAAGATT-3’; antisense: 5’-UCUUUUUUCAUUAAGAAUCTT-3’), *MKRN1* (sense: 5’-GGUGAAGCGGAGUCAAGAA-3’; antisense: 5’-CUUGACUCCGCUUCACCAA-3’) and the corresponding negative control (siNC) were synthesized by GenePharma (Shanghai, China).

### Cells and viruses

PAMs, the natural host cells of PRRSV in vivo, as well as the African green monkey kidney epithelial cell line MA-104 and its derivative MARC-145 cells (a commonly used PRRSV-permissive cell line in vitro), were used as infection models in the present study. For some experiments, immortalized PAMs were used. MARC145 and human embryonic kidney cells (HEK293T) were cultured in Dulbecco’s modified Eagle’s medium (DMEM) supplemented with 10% fetal bovine serum (FBS), 100 U/mL penicillin, and 100 U/mL streptomycin. PAMs were obtained via postmortem lung lavage of 8-week-old PRRSV-negative pigs and cultured in RPMI 1640 medium supplemented with 10% FBS, 100 U/mL penicillin, and 100 U/mL streptomycin. All the cells were maintained in a humidified incubator at 37 °C with 5% CO₂. The animal experiments were performed in strict accordance with the guidelines of the Institutional Animal Care and Use Committee and approved by the Animal Care and Use Committee of Henan Agricultural University (Zhengzhou, Henan, China).

The following PRRSV strains were propagated, titrated in MARC-145 cells, and preserved in our laboratory: GD-HD (GenBank accession no. KP793736.1), SD16 (GenBank accession no. JX087437.1), NADC30-Like (GenBank accession no. MH500776.1), VR2332 (GenBank accession no. EF536003.1), and JXA1 (GenBank accession no. EF112445.1). GD-HD was used in most experiments unless otherwise specified.

### Cell viability and antiviral activity assay

The cytotoxic effect of SalA on MARC-145 cells and PAMs was evaluated using a Cell Counting Kit-8 (CCK-8, C0038, Beyotime Biotechnology) following the manufacturer’s instructions. Briefly, the protocol was as follows: fresh MARC-145 cells (1 × 10^4^ cells/well) or PAMs (1 × 10^5^ cells/well) were seeded into 96-well plates. Approximately 24 h later, the cells were treated with 0, 2.5, 5, 10, 20, 40, 60, 80, or 100 μM SalA for 48 h. Subsequently, 10 μL of CCK-8 reagent was added to each well, and the cells were further cultured for 2 h. Each sample was tested in 5 parallel replicate wells. The optical density (OD) of each well at 450 nm was measured using an automated ELISA plate reader (BioTek; Agilent Technologies, Inc.). The 50% cytotoxic concentration (CC_50_) was calculated using GraphPad Prism 8.0 software on the basis of the OD_450_ values.

To assess the anti-PRRSV activity of SalA in MARC-145 cells and PAMs, the 50% inhibitory concentration (IC_50_) of SalA on PRRSV was determined. MARC-145 cells or PAMs were infected with GFP-PRRSV at a multiplicity of infection (MOI) of 0.1 for 1 h at 37 °C. The cells were subsequently treated with twofold serially diluted SalA in DMEM supplemented with 3% FBS for 36 h, with the maximal noncytotoxic concentration (MNCC) set as the highest concentration. The percentage of GFP-positive cells in each sample was detected by flow cytometry, and the IC_50_ was calculated using GraphPad Prism 8.0 software. The selective index (SI) was calculated as the ratio of the CC_50_ to the IC_50_. The MNCC was defined as the highest concentration of SalA that did not exhibit cytotoxic effects, as detected by the CCK-8 assay.

### Cell treatment

To determine whether SalA affects PRRSV attachment to host cells, MARC-145 cells were seeded into 24-well plates at a density of 5 × 10^4^ cells/well. Approximately 24 h later, when the cells reached 80% confluence, they were precooled at 4 °C for 30 min and then co-incubated with 10 MOI of PRRSV stocks in the presence of 0, 2.5, 5, 10, or 20 µM SalA for 1 h at 4 °C. The viral mixture was discarded, and the cells were washed 3 times with precooled PBS before being harvested to detect PRRSV ORF7 mRNA expression.

For the viral internalization assay, MARC-145 cells were precooled at 4 °C for 30 min and then co-incubated with 10 MOI of PRRSV stocks for 1 h at 4 °C. After being washed twice with precooled PBS, the cells were cultured in DMEM supplemented with 0, 2.5, 5, 10, or 20 µM SalA, transferred immediately to 37 °C, and incubated for an additional 1 h. The cells were harvested to detect PRRSV ORF7 mRNA expression.

To determine whether SalA affects PRRSV replication, MARC-145 cells or PAMs were infected with 0.1 MOI PRRSV and incubated at 37 °C for 1 h. After being washed twice with PBS, the cells were cultured in 3% FBS + DMEM containing 0, 2.5, 5, 10, or 20 µM SalA. At 36 h post-infection (hpi), the cells and supernatants were harvested to detect the expression of the PRRSV N and Nsp4 proteins or the progeny viral titres in the supernatants.

To investigate the impact of SalA on PRRSV-induced inflammatory responses, PRRSV-infected PAMs were treated with 0, 5, 10, or 20 µM SalA starting at 1 hpi. At 36 hpi, the cells were collected to detect the mRNA or protein expression of inflammatory cytokines or proteins in related signalling pathways. Alternatively, PAMs were treated with 1 µg/mL LPS and 0, 5, 10, or 20 µM SalA. At 24 h post-treatment, the cells were harvested to detect inflammatory cytokines or the genes/proteins of related pathways.

### Animal experiments

Twenty 4-week-old healthy piglets were randomly divided into four groups: Mock/HP-PRRSV, SalA treatment (5 mg/kg)/HP-PRRSV, SalA treatment (10 mg/kg)/HP-PRRSV, and Mock control group. The Mock/HP-PRRSV and SalA treatment/HP-PRRSV groups were challenged intranasally with the HP-PRRSV JXA1 strain (10^5^ TCID_50_), and the Mock control group received an equal volume of DMEM. The dosages of SalA used in the in vivo experiments were selected on the basis of previous studies [34, 35].

At 6 h post-challenge (dpc), the SalA treatment/HP-PRRSV groups were intramuscularly injected with SalA (5 mg/kg or 10 mg/kg), and the administration of SalA continued for 3 days. The remaining two groups were intramuscularly injected with an equal volume of DMSO. Piglets were monitored daily for clinical symptoms and rectal temperature. All surviving piglets were sacrificed at 14 dpc. Serum and lung tissues were collected to determine viral loads using TaqMan probe-based qRT-PCR.

Macroscopic lung lesions were examined and scored on the basis of the percentage of infected tissues according to a standard scoring system previously described [36]. Lung tissue samples were fixed in 10% formalin and embedded in paraffin blocks. After the blocks were sectioned, hematoxylin and eosin (H&E) staining was used to observe the histopathological changes under a microscope, and immunohistochemistry was performed using an anti-N mAb.

### RNA sequencing and bioinformatics analysis of transcriptomes

MARC-145 cells were treated with DMSO or 20 μM SalA for 24 h, with three biological replicates per sample. The cells were then collected and resuspended in TRIzol™ reagent for total RNA extraction. RNA quality was assessed via an Agilent 2100 Bioanalyzer (Agilent Technologies, CA, USA) and agarose gel electrophoresis. Next, mRNA were enriched, fragmented, and reverse transcribed into cDNA, which was then sequenced using the Illumina NovaSeq 6000 platform at Ouyi Biomedical Technology Co., Ltd. (Shanghai, China). Differentially expressed genes (DEGs) were analysed using DESeq2. To determine the biological functions of the DEGs, GO enrichment analysis was carried out, covering two functional categories: biological processes and molecular functions. To identify the cellular pathways potentially regulated by SalA, the Kyoto Encyclopedia of Genes and Genomes (KEGG) database was used for further analysis. For the GO analysis, the top 10 significantly enriched biological processes and molecular functions are listed. For the KEGG analysis, the top 20 significantly enriched biological pathways corresponding to each condition in both groups are shown.

### TCID_50_

Following the indicated treatment, the cell culture supernatants were collected, and the viral titres were determined using the Reed‒Muench method. Briefly, the procedure was as follows: MARC-145 cells were plated in 96-well plates at a density of 1 × 10^4^ cells/well. Approximately 24 h later, the supernatants were serially diluted tenfold in DMEM and added to 96-well plates (100 μL/well), with 8 replicates per sample. After incubation at 37 °C for 1 h, the viral solutions were discarded, and the plates were washed with PBS. Subsequently, 175 μL/well of 3% FBS + DMEM was added, and the cells were cultured for 5–7 days. The cytopathic effect (CPE) of each well was observed, and the 50% tissue culture infectious dose (TCID₅₀) was determined using the Reed‒Muench method.

### Cellular thermal shift assay (CETSA)

To determine the effect of SalA on the thermal stability of the Keap1 protein, MARC-145 cells or PAMs were lysed on ice for 30 min in NP-40 lysis buffer supplemented with 1 mM PMSF. After centrifugation at 13 000 × *g* at 4 °C for 15 min, the supernatants were collected. SalA was added to one set of supernatants to a final concentration of 20 μM (designated the SalA-treated group), while the other set of supernatants was treated with an equal volume of DMSO (serving as the control group). Both sets of supernatants were divided into 8 equal aliquots and incubated at 37 °C, 41 °C, 45 °C, 49 °C, 53 °C, 57 °C, 61 °C, and 65 °C for 7 min, respectively. After cooling at room temperature (RT) for 3 min, the mixtures were centrifuged at 13 000 × *g* at 4 °C for 15 min. The supernatants from each sample were then transferred to clean EP tubes, followed by western blotting analysis to determine the Keap1 level.

### Drug affinity responsive target stability (DARTS)

The DARTS assay was performed as follows: MARC-145 cell lysates were incubated with or without 5, 10, or 20 μM SalA at RT for 2 h. Pronase was then added to each sample to a final concentration of 1 μg/mL, followed by incubation at RT for 30 min. Pronase digestion was stopped by the addition of 2 × protein loading buffer, and the samples were boiled at 95 °C for 5 min. Western blotting analysis was then conducted to detect the content of Keap1 protein.

### Small molecule‒protein interaction pull-down assay

Bio-SalA was mixed with 500 μL of PBS containing streptavidin-coated magnetic beads, followed by gentle rotation at RT for 2 h. Free biotin was used as the negative control. Next, the supernatants were discarded, and the remaining beads were incubated with lysates from MARC-145 cells or PAMs at RT for 2 h. After washing three times with PBS, the beads were mixed with 2 × protein loading buffer and boiled at 95 °C for 5 min. The target protein was detected using an anti-Keap1 antibody. Total cell lysates served as the input control.

### Immunofluorescence assay (IFA)

Following treatment, MARC-145 cells or PAMs were fixed at 4 °C for 30 min using prechilled 70% ethanol (stored at −20 °C). After being washed three times with PBS, mouse anti-PRRSV N and Nsp4 protein antibodies (1:300) were added and incubated at RT for 1 h. The cells were then washed three times with PBS and incubated with Alexa Fluor 594-conjugated goat anti-mouse IgG (H&L) (1:500) for 1 h at RT in the dark. Subsequently, 4’,6-diamidino-2-phenylindole (DAPI) was used to stain the cell nuclei. For confocal analysis, the cells were seeded onto glass coverslips placed in 24-well plates prior to treatment. Finally, immunofluorescence images were acquired using a laser scanning inverted confocal microscope equipped with a Zeiss 63 × /1.4 NA oil immersion objective (Zeiss LSM 510, Oberkochen, Germany).

### Quantitative real-time PCR

After MARC-145 cells or PAMs were treated accordingly, total RNA was extracted using TRIzol™ reagent (Invitrogen, Waltham, MA, USA), and cDNA was subsequently obtained from total RNA (500 ng) using a Novoscript® Plus All-in-one 1^st^ Strand cDNA Synthesis SuperMix following the manufacturer’s protocol. The cDNA was used for real-time quantitative reverse transcriptase PCR (qRT-PCR) analysis on an Applied Biosystems QuantStudio system (Thermo Fisher Scientific, Inc.). The expression levels of the target genes were normalized to those of the endogenous reference gene β-actin and quantified via the threshold cycle (2^−ΔΔCt^) method.

### Coimmunoprecipitation (Co-IP) and western blotting analysis

For co-IP analysis, transfected HEK293T cells were lysed on ice in NP-40 lysis buffer containing 1% PMSF for 30 min. The lysates were then centrifuged at 13 000 × *g* at 4 °C for 10 min to completely remove cell debris. Next, the lysates were co-incubated with protein G MagBeads precoated with the corresponding primary antibodies at a dilution of 1:50 overnight at 4 °C on a tube rotator. After gentle washing with ice-cold PBS three times, the beads were directly mixed with 2 × SDS loading buffer, boiled at 95 °C for 5 min, and then centrifuged at 13 000 × *g* at 4 °C for 10 min. The supernatants were collected for subsequent SDS‒PAGE and western blotting analysis.

For western blot analysis of the cell samples, 30 μg of total protein from each sample was loaded and separated via SDS‒PAGE. The target proteins were subsequently transferred to polyvinylidene fluoride (PVDF) membranes, which were subsequently blocked with 5% nonfat dry milk in PBST at 4 °C overnight. Next, the membranes were incubated with primary antibodies against the following targets: anti-Myc-tag (1:1000), anti-HA-tag (1:1000), p-ERK (1:1000), ERK (1:1000), p-P65 (1:1000), P65 (1:1000), p-IκBα (1:1000), IκBα (1:1000), JNK (1:1000), p-JNK (1:1000), P38 (1:1000), p-P38 (1:1000), TLR4 (1:1000), MyD88 (1:1000), Txnip (1:1000), NLRP3 (1:1000), α-tubulin (1:5000), Nrf2 (1:1000), Keap1 (1:1000), IL-1β (1:1000), ASC (1:1000), caspase-1 (1:1000), GSDMD (1:1000), NQO1 (1:1000), N (1:2000) or Nsp4 (1:2000) at RT for 1 h. After washing three times with PBST, the membranes were incubated with HRP-conjugated goat anti-mouse IgG (H&L) and anti-rabbit IgG (H&L) (1:2000 The target bands were visualized via enhanced chemiluminescence (ECL) reagent on an Amersham Imager 600 instrument (Cytiva, Washington D.C., USA).

### Molecular docking

The structure of SalA was determined using the PubChem database (compound CID: 5,281,793) [37]. The 3D structure of the porcine Keap1 protein was predicted using AlphaFold software [38]. To verify the binding site(s) between SalA and its potential target protein Keap1, Molecular Operating Environment 2015 software (MOE 2015, Chemical Computing Group ULC, Montreal, Canada) was used to construct a molecular docking model between SalA and Keap1. The minimum binding energies were calculated and ranked to evaluate the binding stability between SalA and Keap1, and the top-ranked complex was selected for further analysis. The binding energy is inversely proportional to the binding ability, with a binding energy ≤ -5 kcal/mol indicating strong binding activity [39]. The docking results were visualized using PyMOL 2.3.2 (Schrödinger, LLC, New York, USA).

### Molecular dynamic simulation (MDS)

The molecular dynamics simulations were performed using Desmond/Maestro (non-commercial version 2022.1; Schrödinger, LLC), a molecular dynamics software. TIP3P water molecules were added to the systems, which were then neutralized with 0.15 M NaCl solution. After minimization and relaxation of the system, the production simulation was performed for 100 ns in an isothermal-isobaric ensemble at 300 K and 1 bar. The trajectory coordinates were recorded every 100 ps. The molecular dynamics analysis was performed using the Simulation Interaction Diagram from Desmond.

### Statistical analysis

All experiments were conducted independently with at least three biological replicates. The results obtained were consistent, and typical images are presented. For the analysis of statistical significance, GraphPad Prism 8.0 software (GraphPad Software, Inc., San Diego, USA) was used. Two groups were compared by using the unpaired Student’s *t* test, and multiple groups were compared by one-way analysis of variance (ANOVA). All the data were normalized to the control values of each assay and are presented as the mean ± standard deviation (SD). A *P* value < 0.05 was considered statistically significant.

## Results

### SalA inhibits PRRSV replication in vitro

**Figure 1:**
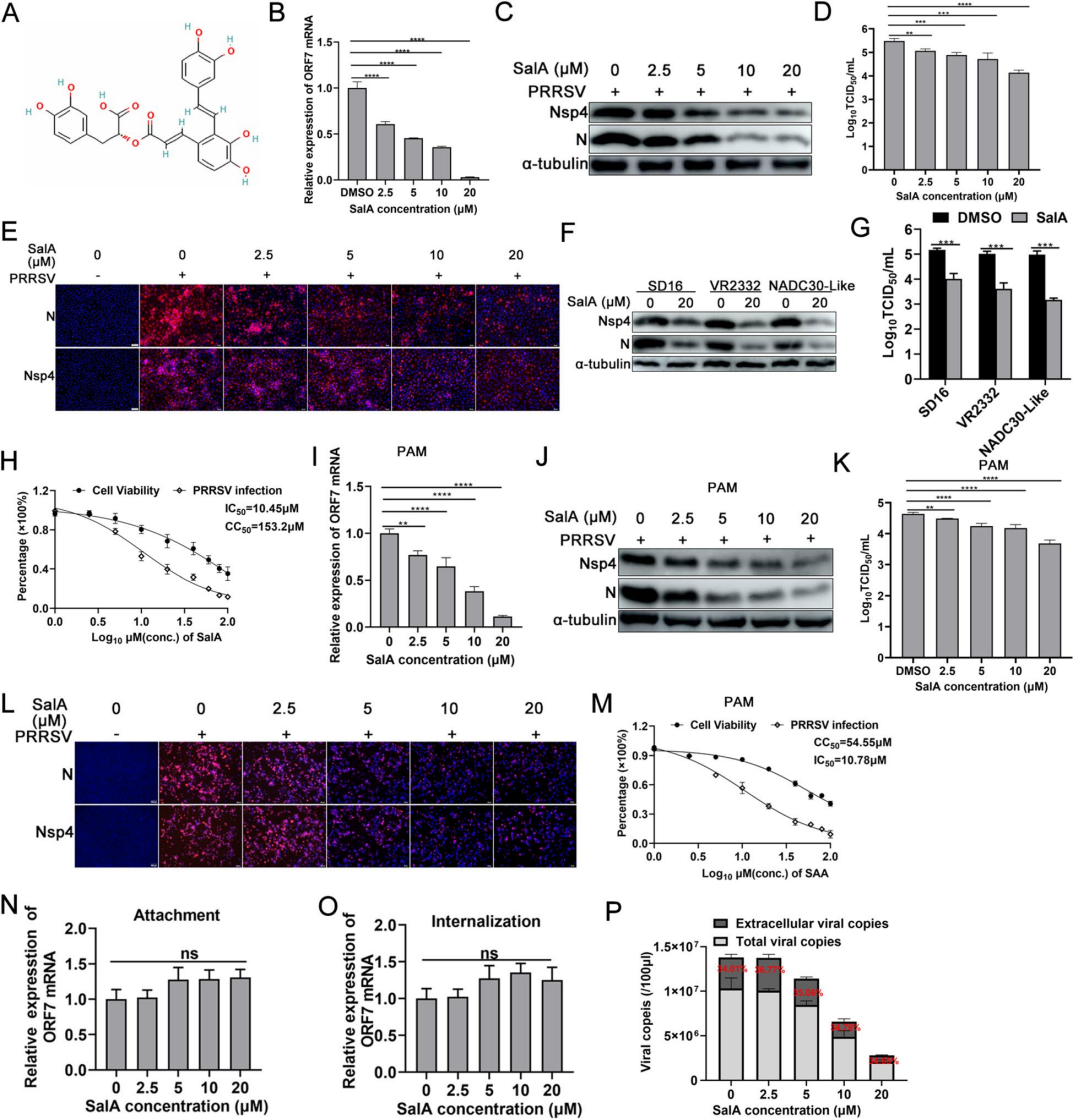
**SalA suppresses PRRSV replication in vitro.** **A** The chemical structure of SalA. MARC-145 cells were infected with 0.1 MOI PRRSV. At 1 hpi, the cells were cultured with 3% FBS + DMEM containing 0, 2.5, 5, 10, or 20 μM SalA. The cells were harvested at 36 hpi to detect the expression of ORF7 mRNA by qRT-PCR (**B**), the N and Nsp4 proteins using western blotting (**C**), or IFA (E); the supernatant viral titres were detected using TCID_50_ (**D**). **F** and **G** MARC-145 cells infected with 0.1 MOI of SD16, VR2332, or NADC30-like PRRSV were collected at 36 hpi. The cells were used for detection of PRRSV N and Nsp4 protein expression using western blotting (**F**), and the supernatants were used for detection of viral titres using TCID_50_ (**G**). **H** The CC_50_ value of SalA in MARC-145 cells and the IC_50_ value of SalA for PRRSV. **I**–**L** PAMs were infected with 0.01 MOI PRRSV. At 1 hpi, the cells were further cultured with 3% FBS + DMEM containing 0, 2.5, 5, 10, or 20 μM SalA. At 24 hpi, the cells and culture supernatants were collected for the analysis of PRRSV ORF7 mRNA using qRT-PCR (**I**), N and Nsp4 protein expression using western blotting (**J**), viral titres using TCID_50_ (**K**), and N and Nsp4 protein expression using IFA (**L**). (M) The CC_50_ value of SalA in PAMs and the IC_50_ value of SalA in PRRSV. **N** Effect of SalA treatment on PRRSV attachment to MARC-145 cells. **O** Effect of SalA treatment on PRRSV entry into MARC-145 cells. **P** Effects of SalA treatment on PRRSV release from MARC-145 cells.

The chemical structure of SalA is shown in Figure 1A. To determine the effect of SalA on PRRSV replication, MARC-145 cells infected with 0.1 MOI of PRRSV were treated with various doses of SalA starting at 1 hpi. SalA significantly suppressed the expression of PRRSV ORF7 mRNA (Figure 1B) and the N and Nsp4 proteins in a dose-dependent manner (Figure 1C). A TCID₅₀ assay was performed to detect supernatant progeny viral titres. Compared with those in the DMSO treatment group, the supernatant viral titres decreased by 0.42 log_10_, 0.6 log_10_, 0.77 log_10_, and 1.35 log_10_, respectively, with increasing SalA concentration (Figure 1D). The IFA results confirmed that PRRSV N and Nsp4 protein expression also exhibited a similar dose-dependent decreasing trend with SalA treatment (Figure 1E).

Next, we investigated whether the inhibitory effect of SalA on PRRSV replication was strain dependent. MARC-145 cells were infected with 0.1 MOI of SD16, VR2332, or NADC30-Like PRRSV and then treated with 20 μM SalA. The western blotting results revealed that SalA markedly suppressed the expression of the N and Nsp4 proteins in all three PRRSV strains (Figure 1F). TCID₅₀ analysis of the supernatant viral titres revealed that, compared with the control treatment, SalA treatment reduced the viral titre by 1.16 log_10_, 1.4 log_10_, and 1.81 log_10_, respectively (Figure 1G). To exclude the possibility that the ability of SalA to suppress PRRSV replication was attributed to its cytotoxic effect, the CC₅₀ of SalA in MARC-145 cells was determined. The results revealed that the CC₅₀ of SalA in MARC-145 cells was 153.2 μM, the IC₅₀ value of SalA against PRRSV was 10.45 μM, and the selectivity index (SI) of SalA against PRRSV in MARC-145 cells was 14.66 (Figure 1H).

PAMs are the primary natural host cells of PRRSV, so further assessment of the anti-PRRSV activity of SalA in PAMs is important. PAMs were infected with 0.01 MOI of PRRSV and then treated with different concentrations of SalA. The qRT-PCR results revealed that SalA treatment dose-dependently decreased the PRRSV ORF7 mRNA level (Figure 1I), and the western blotting results revealed reduced expression of the PRRSV N and Nsp4 proteins (Figure 1J). TCID₅₀ analysis revealed that, compared with the control treatment, SalA treatment decreased the supernatant viral titre by 0.14 log_10_, 0.39 log_10_, 0.45 log_10_, and 0.95 log_10_, respectively (Figure 1K). IFA yielded results similar to those of western blotting (Figure 1L). The CC₅₀ of SalA in PAMs was 54.55 μM, the IC₅₀ of SalA against PRRSV was 10.78 μM, and the selectivity index (SI) of SalA against PRRSV in PAMs was 5.06 (Figure 1M).

To clarify which step of the PRRSV life cycle is affected by SalA, viral attachment and internalization experiments were conducted. The qRT‒PCR results revealed that SalA treatment affected neither PRRSV binding to host cells (Figure 1N) nor PRRSV entry into host cells (Figure 1O). For viral release analysis, representative results revealed that extracellular viral copies account for approximately 34.01%, 36.77%, 35.06%, 34.78%, and 34.65% of the total number of viral copies (intracellular + extracellular) in the 0, 2.5, 5, 10, and 20 μM SalA groups, respectively (Figure 1P). Furthermore, the results from different batches of experiments demonstrated that the proportions of extracellular viral copies relative to total viral copies did not differ significantly among the groups, with no interbatch variation in the experimental data (*P* > 0.05, data not shown), indicating that SalA treatment did not significantly affect viral release. Taken together, these results suggest that SalA suppresses PRRSV replication in vitro.

### SalA treatment inhibits viral replication in piglets

**Figure 2:**
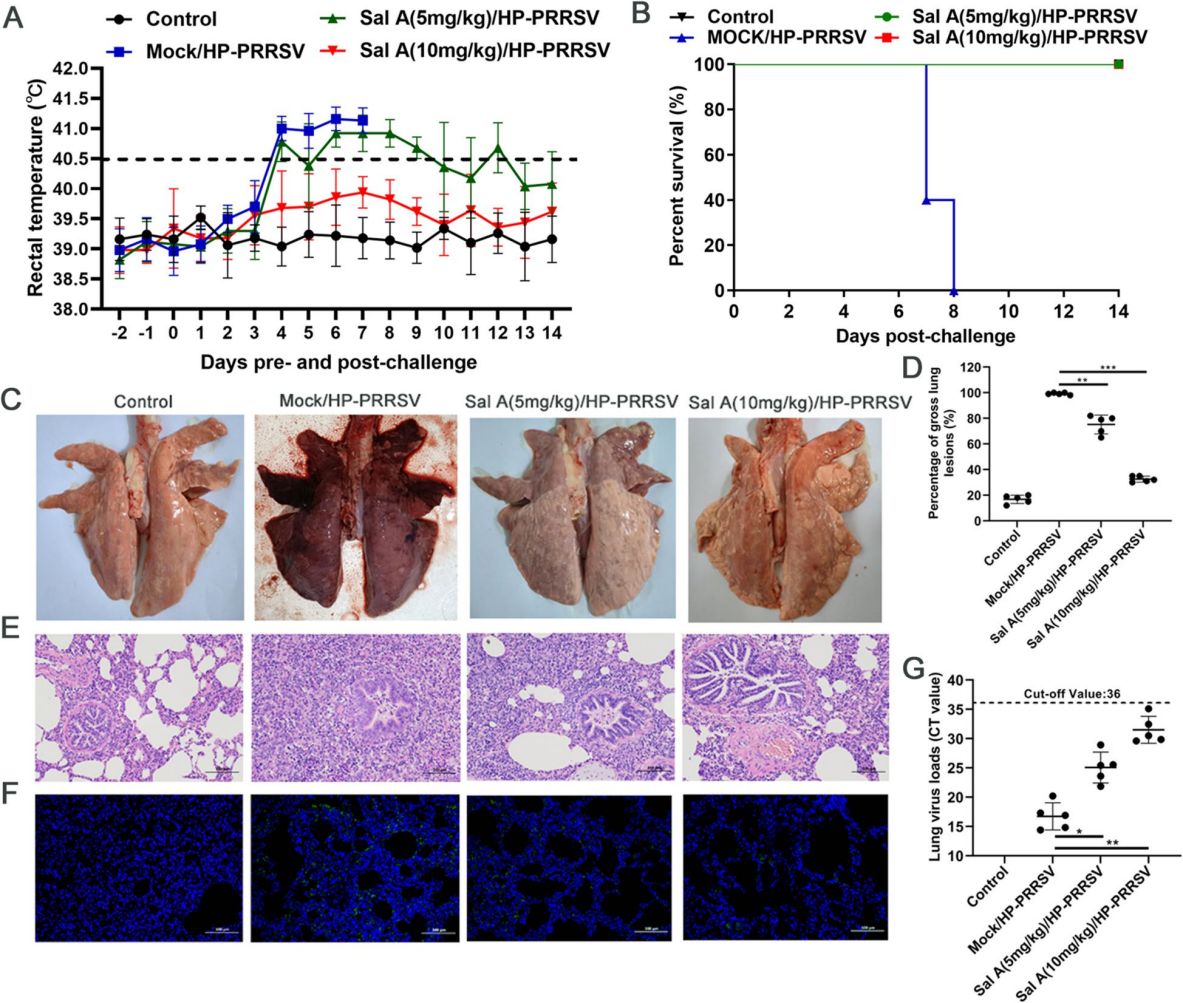
**SalA treatment can inhibit HP-PRRSV JXA1 strain replication in piglets and reduce the number of lung lesions.** **A** Rectal temperature was recorded daily for all groups. A temperature ≥ 40.5 °C was defined as a clinical fever. **B** Survival curves of the piglets in each group (*n* = 5). **C** Gross lesions of pigs’ lungs. For each group, representative images were captured immediately after piglets were autopsied at death or 14 dpc. **D** The gross lung lesion scores of each piglet were calculated on the basis of the percentage of lung area affected. **E** Microscopy image of lung lesions. Scale bar: 100 μm. **F** Immunohistochemical staining of the lungs. Scale bar: 100 μm. **G** Viral RNA loads in the lung tissue of each pig were measured using TaqMan probe-based qRT-PCR.

On the 4^th^ day after the challenge, the rectal temperature of all pigs in the challenge control group developed a high fever (≥ 40.5 °C) and presented a range of clinical symptoms, including dyspnea, cough, loss of appetite and listlessness. On the 4^th^ day after the challenge, all pigs in the 5 mg/kg SalA treatment group also developed a high fever (≥ 40.5 °C), with a range of clinical symptoms such as dyspnea, cough, and loss of appetite; however, their temperature returned to normal by the 11^th^ dpc. On the 4^th^ day after the challenge, 2 out of the 5 pigs in the 10 mg/kg SalA treatment group presented a temperature increase to 40.2 °C but no typical clinical symptoms, with temperatures returning to normal on the 7^th^ dpc (Figure 2A). All piglets in the challenge group died between 7 and 8 dpc, whereas those in the treatment groups survived. In the control group, the rectal temperature remained normal throughout the experimental period, and no deaths had occurred (Figures 2A and 2B).

At 14 dpc, all surviving pigs were sacrificed, and pathological changes in the lungs were observed. The lungs of the Mock/HP-PRRSV group presented severe pulmonary edema, hemorrhage, and consolidation. The 5 mg/kg SalA treatment group also exhibited pulmonary edema, hemorrhage, and partial consolidation, but the degree of lesions was milder than that in the Mock/HP-PRRSV group. In contrast, lungs from the 10 mg/kg SalA treatment group exhibited only mild congestion (Figure 2C).

To better quantify these pathological changes, a gross lung lesion scoring system was used. The gross lung lesion scores of the piglets in the Mock/HP-PRRSV group were significantly greater than those in the SalA treatment/HP-PRRSV group (Figure 2D). Additionally, the scores of the 5 mg/kg SalA treatment/HP-PRRSV group were significantly greater than those of the 10 mg/kg SalA treatment/HP-PRRSV group (Figure. 2D). Pigs in the mock control group presented no gross lung lesions.

Compared with those of the control group, the HP-PRRSV infection group presented moderate parenchymal transformation in the lung tissue, including alveolar structure loss, mild compensatory alveolar expansion, extensive interstitial granulocyte infiltration, and abundant necrotic cell debris. In the 5 mg/kg SalA treatment/HP-PRRSV group, numerous lymphocytes and granulocytes infiltrated the alveolar walls, which was accompanied by severe alveolar wall thickening, widened alveolar septa, and dilation of many alveoli. Additionally, shed epithelial cells and eosinophilic substances could be observed in the lumina of local bronchioles. In the 10 mg/kg SalA treatment/HP-PRRSV group, some alveolar walls thickened, diffuse granulocyte infiltration into the alveolar walls, and mild interstitial vascular congestion were observed. Lung pathological damage in the SalA-treated/HP-PRRSV groups was milder than that in the Mock/HP-PRRSV group, with the 10 mg/kg SalA treatment group showing less severe injury than the 5 mg/kg SalA treatment group (Figure 2E). Immunohistochemical analysis revealed that the fluorescence intensity of the viral N protein in the mock/HP-PRRSV group was significantly greater than that in the treatment groups, that in the 5 mg/kg SalA treatment group was greater than that in the 10 mg/kg SalA treatment group, and no fluorescence was observed in the control group (Figure 2F).

Additionally, viral loads in the lungs were quantified via qRT‒PCR with TaqMan probes. Compared with those in the mock/HP-PRRSV group and the 5 mg/kg SalA treatment group, the CT values of viral RNA in the 10 mg/kg SalA treatment group were reduced by 14 and 5 CT values, respectively (*P* < 0.05, Figure 2G). Collectively, these findings indicate that SalA treatment effectively mitigates lung injury caused by infection with the HP-PRRSV JXA1 strain in pigs.

### RNA transcriptome sequencing indicates that SalA treatment activates the Keap1-Nrf2-NQO1 signalling pathway

**Figure 3:**
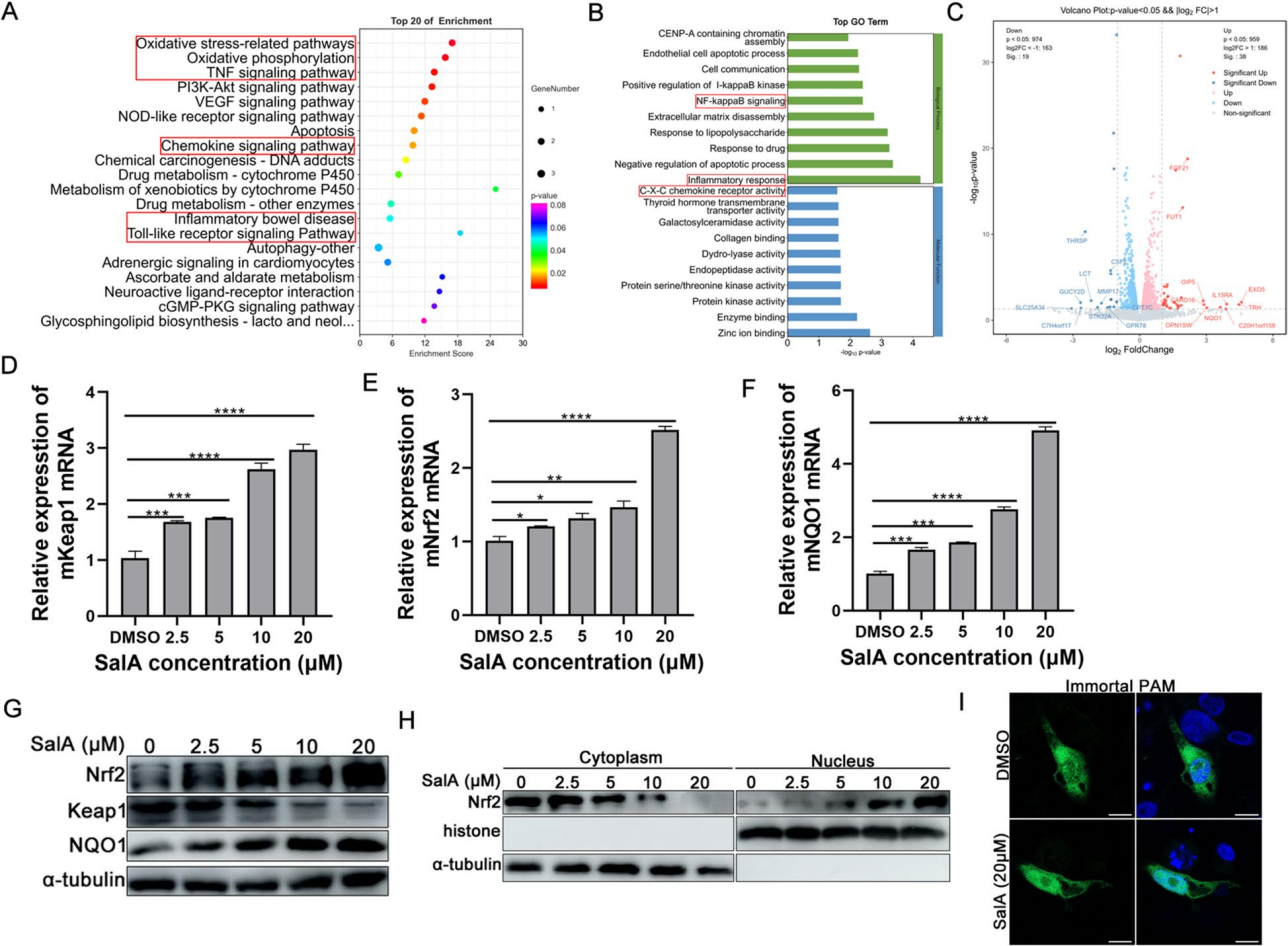
**Transcriptional response of MARC-145 cells to SalA treatment.** RNA profiling by RNA sequencing of MARC-145 cells treated with 20 µM SalA or DMSO for 24 h. **A** KEGG pathway enrichment analysis of potential pathways regulated by SalA. The color gradient represents the P value, and the size of the black spots represents the number of genes enriched in each pathway. **B** GO analysis of the DEPs in terms of biological process and molecular function. **C** Volcano plot showing upregulated (red) and downregulated (blue) genes between the DMSO control and SalA treatment groups. **D**–**G** MARC-145 cells were treated with 0, 2.5, 5, 10, or 20 μM SalA for 24 h. The cells were harvested, and the mRNA levels of Keap1 (**D**), Nrf2 (**E**), and NQO1 (**F**) were measured by qRT-PCR. The protein expression of Keap1, Nrf2, and NQO1 was analysed using western blotting (**G**). **H** Cytoplasmic and nuclear extracts of PAMs treated with 0, 2.5, 5, 10, or 20 μM SalA for 24 h were prepared, and western blotting was performed to analyse the subcellular distribution of Nrf2. **I** Confocal analysis of the subcellular localization of Nrf2 in immortalized PAMs after treatment with DMSO or 20 μM SalA for 24 h.

To further clarify the mechanism underlying the anti-PRRSV activity of SalA, MARC-145 cells treated with DMSO or 20 μM SalA were subjected to high-throughput transcriptome sequencing. KEGG pathway enrichment analysis of the transcriptomic data revealed that SalA treatment significantly regulated oxidative stress-related pathways, chemokine pathways, and TLR pathways (Figure 3A). GO enrichment analysis further revealed that SalA affected the NF-κB signalling pathway and inflammatory response processes (Figure 3B). The volcano plot revealed an increase in the *NAD(P)H:quinone oxidoreductase 1* (*Nqo1*) gene (product: NQO1) among the numerous genes upregulated upon SalA exposure (Figure 3C). According to the literature, *Nqo1* is one of the target genes of the transcription factor Nrf2 [40, 41]. Under stress or cytotoxic stimulation, Nrf2 dissociates from the E3 ubiquitin ligase Keap1 to avoid ubiquitination and subsequent degradation. It then translocates to the nucleus and activates a collection of genes, such as *heme oxygenase 1* and *Nqo1*, to combat oxidative stress and inflammation [42, 43]. Considering the results of the GO enrichment, KEGG analysis, and DEG analyses, subsequent experiments focused on the Nrf2-mediated pathway to clarify the mechanism by which SalA inhibits PRRSV replication.

To validate whether Nrf2-related pathways were indeed affected by SalA, MARC-145 cells were treated with the indicated doses of SalA for 24 h. Subsequently, the transcriptional levels of *Keap1*, *Nrf2*, and *Nqo1* were measured by qRT-PCR. The results suggested that, compared with those in the control group, the levels of the *Keap1* (Figure 3D), *Nrf2* (Figure 3E), and *Nqo1* (Figure 3F) mRNA were increased in a concentration-dependent manner. Western blotting was employed to further confirm the qRT-PCR results. The results indicated that treatment of MARC-145 cells with SalA dose-dependently upregulated Nrf2 and NQO1 protein expression (Figure 3G). In contrast to the increase in Keap1 mRNA expression, Keap1 protein levels gradually decreased (Figure 3G).

To further demonstrate the activation of the Nrf2 pathway, the nuclear translocation of Nrf2 in PAMs after SalA treatment was determined. As shown in Figure 3H, SalA treatment concentration-dependently promoted the nuclear translocation of Nrf2. Confocal microscopy analysis of the subcellular distribution of Nrf2 in immortalized PAMs following SalA treatment revealed similar results (Figure 3I).

### SalA promotes Keap1 proteasomal degradation

**Figure 4:**
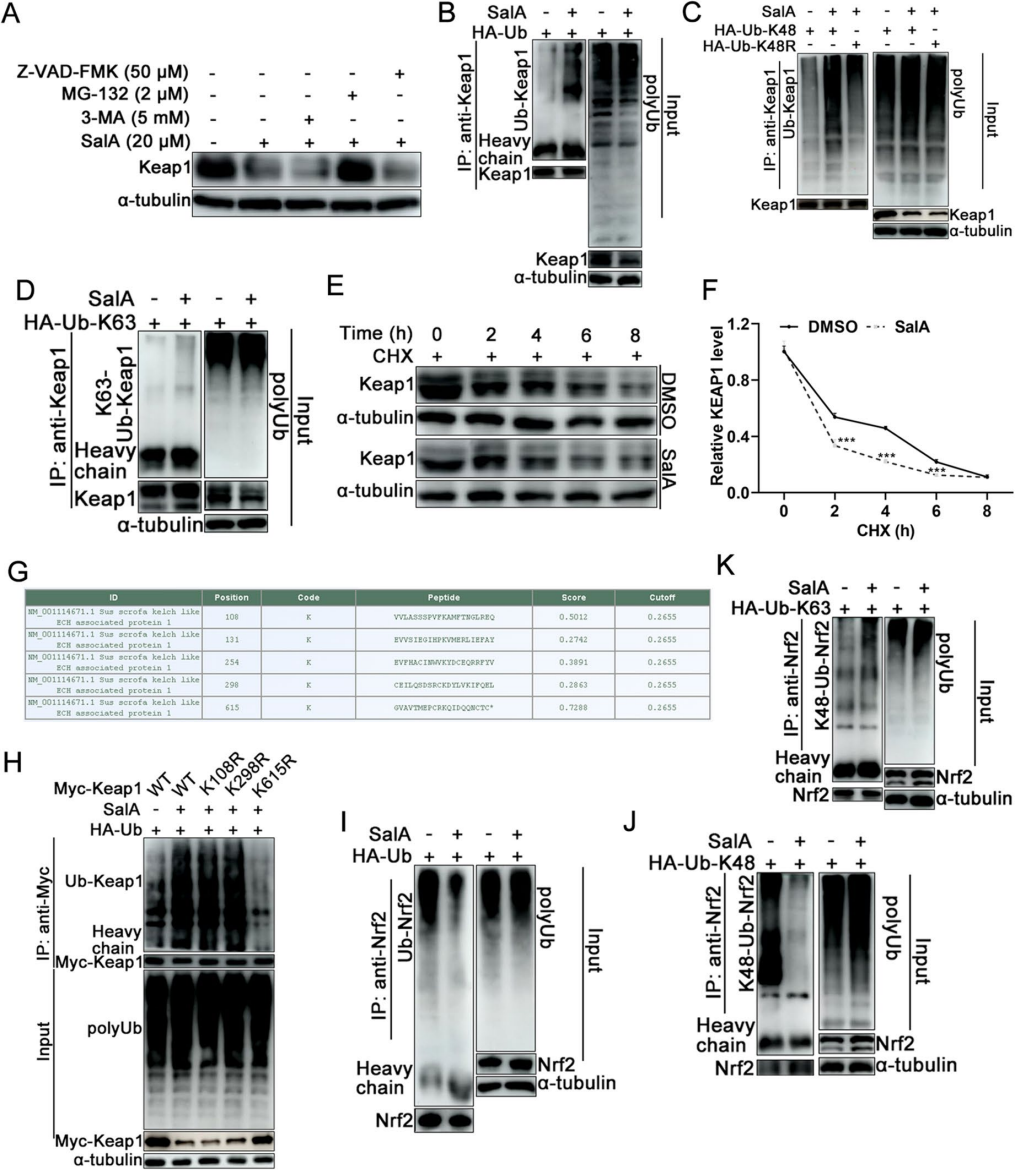
**SalA promotes Keap1 K48-linked ubiquitin-dependent proteasomal degradation.** **A** MARC-145 cells were treated with SalA (20 μM) simultaneously with 3-MA (5 mM), MG132 (2 μM) or Z-VAD-FMK (50 μM) for 24 h. The cells were collected for analysis of Keap1 expression by western blotting. **B**–**D** MARC-145 cells were transfected with HA-Ub, HA-Ub-K48, or HA-Ub-K63 expression plasmids. At 24 hpt, the cells were incubated in medium with or without 20 μM SalA for an additional 24 h. Then, the cells were harvested, and co-IP was conducted to measure the levels of Ub-Keap1 (**B**), K48-Ub-Keap1 (**C**), and K63-Ub-Keap1 (**D**). **E** Levels of Keap1 in MARC-145 cells treated with 50 mg/mL CHX in the presence or absence of DMSO or 20 μM SalA for 0, 2, 4, 6, or 8 h. **F** Relative band intensities of Keap1 in **E** normalized to those of α-tubulin. **G** The potential ubiquitination site(s) of porcine Keap1 were predicted via the online software GPS-Uber. **H** HA-Ub was transfected into HEK293T cells together with Myc-Keap1-WT, Myc-Keap1-K108R, Myc-Keap1-K298R, or Myc-Keap1-K615R, followed by treatment with 20 μM SalA. The cells were collected and then subjected to co-IP analysis. **I**–**K** Effects of SalA treatment on the ubiquitination (**I**), K48-linked ubiquitination (**J**), and K63-linked ubiquitination (**K**) of Nrf2 in MARC-145 cells.

To investigate the pathway involved in Keap1 degradation induced by SalA treatment (Figure 3G), MARC-145 cells were treated with Z-VAD-FMK, MG-132, or 3-MA in combination with SalA or with SalA alone. The western blotting results revealed that SalA promoted Keap1 degradation, but this effect was significantly reversed only by the ubiquitin–proteasome pathway inhibitor MG-132, not by the autophagy inhibitor 3-MA or the apoptosis inhibitor Z-VAD-FMK (Figure 4A). Next, whether SalA promoted Keap1 ubiquitin‒proteasome-mediated degradation by enhancing its polyubiquitination was investigated. MARC-145 cells transfected with the HA-ubiquitin plasmid were treated with or without 20 μM SalA, followed by co-IP analysis to detect Keap1 ubiquitination. SalA markedly promoted the ubiquitination of Keap1 (Figure 4B). K48-linked polyubiquitination typically targets proteins for proteasomal degradation, whereas K63-linked polyubiquitination is associated mainly with autophagy [44–46]. Further analysis revealed that SalA treatment markedly enhanced the K48-linked ubiquitination of Keap1 without significantly affecting its K63-linked ubiquitination (Figures 4C and 4D).

Cycloheximide (CHX), a known de novo protein synthesis inhibitor, was used to evaluate the stability of the Keap1 protein. Compared with DMSO treatment alone, treatment with 20 μM SalA significantly shortened the half-life of the Keap1 protein in MARC-145 cells (Figures 4E and 4F). To further screen and identify the ubiquitination site(s) of Keap1 induced by SalA, the online ubiquitination site prediction software GPS-Uber was used [47]. Porcine Keap1 has five putative lysine (K) ubiquitination sites (Figure 4G), and human Keap1 has ten putative K ubiquitination sites (data not shown). Among these putative ubiquitination sites, K108, K131, K254, K298, and K615 are conserved in both porcine and human Keap1 proteins. On the basis of the prediction system’s ranking, three predicted ubiquitination sites (K108, K298, and K615) of Keap1 were mutated to arginine (R), resulting in K108R, K298R, and K615R mutants, respectively, to identify the site(s) involved in SalA-induced ubiquitination. As shown in Figure 4H, the K108R and K298R mutations did not affect Keap1 ubiquitination, whereas the K615R mutation significantly abolished SalA-induced Keap1 ubiquitination, indicating that SalA treatment enhances Keap1 polyubiquitination at K615.

Since Keap1 negatively regulates Nrf2 by promoting its ubiquitination and degradation, whether SalA treatment affects Nrf2 ubiquitination was further determined. As shown in Figure 4I, SalA treatment significantly reduced total Nrf2 polyubiquitination and K48-linked Nrf2 polyubiquitination (Figure 4J) but slightly promoted K63-linked Nrf2 polyubiquitination (Figure 4K).

### SalA serves as a Keap1 degrader by recruiting the E3 ligase MKRN1 to the ubiquitin‒proteasome system

**Figure 5:**
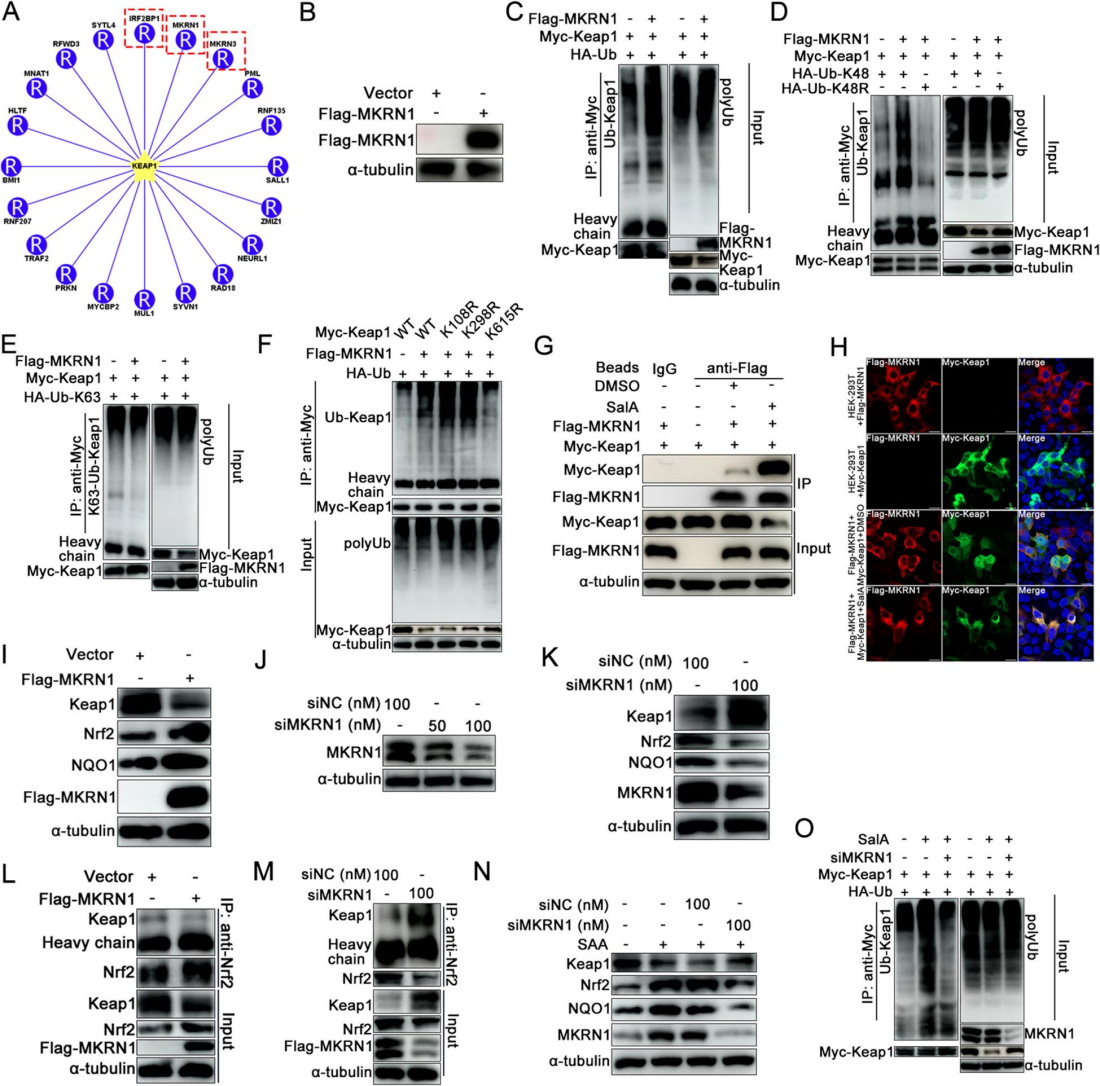
**SalA promotes Keap1 proteasomal degradation by recruiting the E3 ligase MKRN1.** **A** The potential E3 ligases associated with porcine Keap1 ubiquitination were predicted using the online tool UbiBrowser. **B** Identification of MKRN1 expression in HEK293T cells by western blotting. **C**–**E** HEK293T cells were cotransfected with Flag-MKRN1, Myc-Keap1, and either HA-Ub, HA-Ub-K48, HA-Ub-K48R, or HA-Ub-K63 for 48 h. Then, cells were harvested for analysis of the ubiquitination of Keap1 using co-IP assay. **F** Ubiquitination site(s) of Keap1 recognized by the E3 ligase MKRN1. **G** and **H** Co-IP and confocal microscopy were used to validate the interaction between MKRN1 and Keap1 in the presence or absence of SalA. **I** MARC-145 cells were transfected with empty vector or Flag-MKRN1 plasmids. The expression of Keap1, Nrf2, and NQO1 was detected by western blotting. **J** Verification of the MKRN1-specific siRNA knockdown efficiency. **K** Effects of MKRN1 knockdown on the expression of Keap1, Nrf2, and NQO1 in MARC-145 cells. **L** Effect of MKRN1 overexpression on the Keap1‒Nrf2 interaction in MARC-145 cells. **M** Effect of MKRN1 knockdown on the Keap1‒Nrf2 interaction in MARC-145 cells. **N** Effects of MKRN1 knockdown on SalA-induced Keap1, Nrf2, and NQO1 expression. **O** Effect of MKRN1 knockdown on SalA-induced Keap1 ubiquitination.

As SalA promoted Keap1 proteasomal degradation by promoting its K48-linked ubiquitination, it was hypothesized that SalA acts by enhancing the interaction between a specific E3 ubiquitin ligase and Keap1, thereby facilitating Keap1 ubiquitination and degradation. A comprehensive database for proteome-wide known and predicted ubiquitin ligase-substrate interactions in eukaryotic species was utilized to predict the possible E3 ligases participating in porcine Keap1 ubiquitination [48]. According to the rating results, porcine IRF2BP1, MKRN1, and MKRN3 ranked in the top 3 and were most likely to catalyze the ubiquitination of Keap1 (Figure 5A). Thus, recombinant eukaryotic expression plasmids for IRF2BP1, MKRN1, and MKRN3 were constructed and transfected to test their effects on Keap1 ubiquitination, and all the proteins were successfully expressed (Figure 5B and Additional files 1A and C). Ubiquitination analysis revealed that MKRN1 (Figure 5C) effectively catalyzed Keap1 ubiquitination, whereas MKRN3 had a weak effect and that IRF2BP1 had no effect (Additional file 1D). Next, the type of ubiquitination catalyzed by MKRN1 on Keap1 was further determined. MKRN1 promoted the K48-linked rather than the K63-linked ubiquitination of Keap1 (Figures 5D and 5E). MKRN1 catalyzed the ubiquitination of Keap1 at the K615 site (Figure 5F), which was consistent with SalA-induced Keap1 ubiquitination (Figure 4I).

To validate whether SalA acts as an MKRN1-dependent Keap1 degrader, HEK293T cells were co-transfected with the Myc-Keap1 plasmid and empty vector or the Flag-MKRN1 plasmid. At 24 h post-transfection (hpt), the cells were treated with or without SalA. Co-IP revealed a weak interaction between Keap1 and MKRN1 when the cells were treated with DMSO, whereas Keap1 strongly interacted with MKRN1 after SalA treatment (Figure 5G). Furthermore, the confocal microscopy results revealed that Myc-Keap1 colocalized markedly with Flag-MKRN1 in the presence of SalA (Figure 5H).

Next, the effect of MKRN1 on the Keap1-Nrf2-NQO1 pathway was investigated. The overexpression of MKRN1 suppressed Keap1 expression while increasing the expression of both Nrf2 and NQO1 (Figure 5I). A specific siRNA targeting MKRN1 was then synthesized to knock down its expression (Figure 5J). Knockdown of MKRN1 promoted Keap1 expression but inhibited the expression of both Nrf2 and NQO1 (Figure 5K). MKRN1 overexpression weakened the interaction between Keap1 and Nrf2 (Figure 5L), whereas MKRN1 knockdown had the opposite effect (Figure 5M). MKRN1 knockdown partially reversed SalA-induced Keap1 degradation and the upregulation of Nrf2 and NQO1 (Figure 5N). Additionally, knockdown of MKRN1 partially reversed SalA-induced Keap1 ubiquitination (Figure 5O). These results indicate that SalA promotes Keap1 degradation, thereby activating the Nrf2-NQO1 pathway, by recruiting the E3 ligase MKRN1.

### SalA directly binds to the Thr560 residue of Keap1

**Figure 6:**
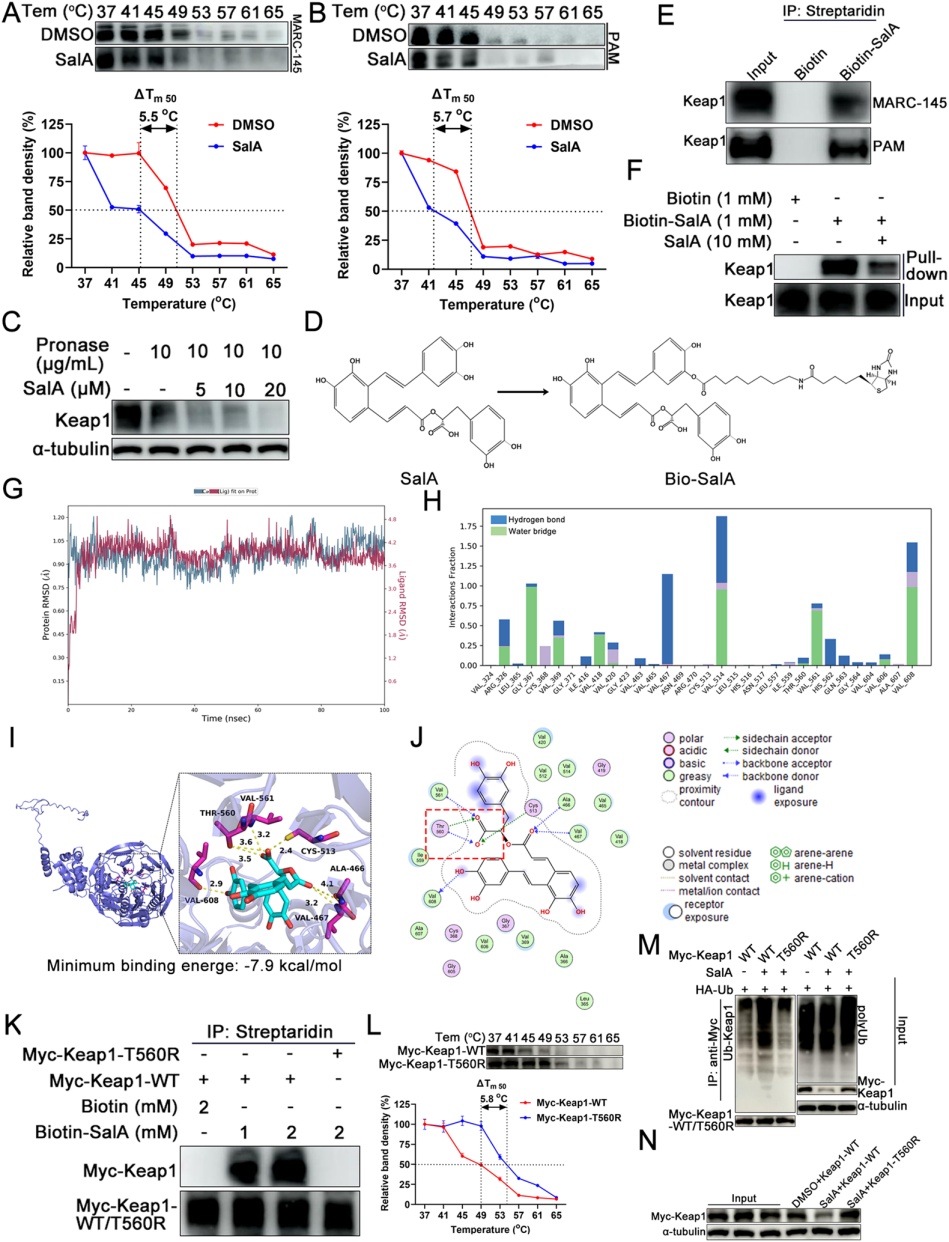
**SalA directly binds to Keap1.** **A** and **B** Western blot-coupled CETS was performed on MARC-145 cells (**A**) and PAMs (**B**), as described in the Materials and Methods. **C** DARTS was performed on MARC-145 cells. **D** Synthesis of the biotinylated probe Bio-SalA. **E** MARC-145 cell or PAM lysates were incubated with biotin (1 mM, control) or bio-SalA (1 mM) in the absence or presence of 10 mM unlabelled SalA to perform a competitive pull-down assay. The precipitates were separated by SDS-PAGE and detected by western blotting with an anti-Keap1 antibody. **F** A biotin-streptavidin pull-down assay using bio-SalA or biotin (control) was performed with lysates from MARC-145 cells. **G** RMSD plot of Keap1 (dark red curve) and SalA (light blue). unit: Å **H** Formation frequency of hydrogen bonds and water bridges mediating the interaction between Keap1 and SalA during MDS. **I** and **J** Docked conformation of SalA with Keap1. SalA and Keap1 are represented as sticks and cartoons, respectively. SalA is colored cyan, and Keap1 is colored magenta to indicate its binding sites. The binding site is shown as a cavity structure. **K** HEK293T cells were transfected with Myc-Keap1-WT or Myc-Keap1-T560R, after which the cell lysates were incubated with biotin (2 mM, control) or bio-SalA (1 and 2 mM). The precipitates were separated by SDS-PAGE and detected by western blotting with an anti-Myc antibody. **L** MARC-145 cells were transfected with Myc-Keap1-WT or Myc-Keap1-T560R. At 36 hpt, the cells were collected, and WB-CETS was carried out to compare the thermal stability of Myc-Keap1-WT and T560R. **M** Effect of SalA on the ubiquitination of Myc-Keap1-T560R. **N** Effect of SalA on the degradation of Myc-Keap1-T560R.

Since SalA promoted Keap1 K48-linked ubiquitination and subsequent proteasomal degradation by recruiting the E3 ligase MKRN1 (Figures 4 and 5), whether SalA directly interacted with Keap1 was further investigated by western blot-coupled CETSA (WB-CETSA). Upon treatment with SalA, Keap1 in MARC-145 cells exhibited a substantial decrease in thermal stability, with a Tm50 (temperature at which 50% of the protein is denatured) decrease of 5.5 °C (Figure 6A). A similar reduction in Keap1 thermal stability was also observed in PAMs after treatment with SalA, with a Tm50 decrease of 5.7 °C (Figure 6B). In addition to the CETSA results, the DARTS assay results revealed that SalA effectively accelerated the pronase-mediated digestion of Keap1 in a dose-dependent manner (Figure 6C).

A biotin-labelled SalA pull-down assay was subsequently used to detect the direct interaction between SalA and Keap1. Biotin-labelled SalA (bio-SalA) was synthesized first (Figure 6D). The results from the biotin-streptavidin pull-down assay demonstrated that bio-SalA directly bound to Keap1 in the lysates of both PAMs and MARC-145 cells (Figure 6E). A competitive pull-down assay revealed that bio-SalA effectively pulled down Keap1, whereas the addition of SalA partially reduced the interaction between bio-SalA and Keap1 (Figure 6F), indicating a competitive relationship between bio-SalA and SalA in binding to Keap1.

A 100 ns MDS was conducted to simulate the protein‒ligand complex, further investigating the dynamic properties of the SalA‒Keap1 complex. The plot revealed that the RMSD of Keap1 remained relatively stable, ultimately stabilizing at approximately 0.9–1.05 Å, whereas the RMSD of SalA remained stable at 3.6–4.2 Å after initial fluctuations (Figure 6G). These results indicated that the initial conformational fluctuations of Keap1 and SalA were relatively small and that the stability of their complex conformations was notably high. The interaction between Keap1 and SalA was monitored throughout the simulation. The schematic diagram shows that multiple hydrogen bonds and water bridges formed between SalA and Keap1 during the simulation (Figure 6H). In summary, the simulation results demonstrate that Keap1 and SalA exhibit high affinity in this conformation.

To further clarify the binding site(s) of SalA to the Keap1 protein, MOE software was used to simulate the binding mode of SalA through molecular docking. SalA was found to form a stable complex with Keap1, as the lowest binding energy of the SalA-Keap1 complex was -7.9 kcal/mol (≤ -5 kcal/mol, indicating strong binding; Figures 6I and 6J). Among the predicted binding sites, SalA most likely bound to Thr560 on Keap1, as two hydrogen bonds formed between the SalA carboxyl group and Keap1 Thr560 (Figures 6I and 6J). To confirm whether SalA bound to Keap1 at Thr560, a recombinant expression plasmid, Myc-Keap1-T560R, was constructed and transfected into HEK293T cells. Biotin-mediated pull-down assays revealed that Myc-Keap1-T560R did not interact with bio-SalA, whereas Myc-Keap1-WT was still pulled down by bio-SalA (Figure 6K). CETSA was further used to evaluate the binding capacity between Myc-Keap1-T560R and SalA. The results showed that the thermal stability of Myc-Keap1-T560R was notably greater than that of Myc-Keap1-WT when Myc-Keap1-T560R was treated with SalA, with a Tm50 increase of 5.8 °C (Figure 6L). Furthermore, the effect of SalA on the K48-linked ubiquitination of Myc-Keap1-T560R was also evaluated. SalA treatment significantly promoted K48-linked ubiquitination of Myc-Keap1-WT; however, the same phenomenon was not observed for Myc-Keap1-T560R (Fig. 6M). SalA treatment degraded Myc-Keap1-WT but not Myc-Keap1-T560R (Figure. 6N). Taken together, these results suggest that SalA promotes Keap1 recruitment to MKRN1 for proteasomal degradation by directly binding to the Thr560 residue of Keap1.

### SalA suppresses PRRSV replication by activating the MKRN1/Nrf2/NQO1 pathway

**Figure 7:**
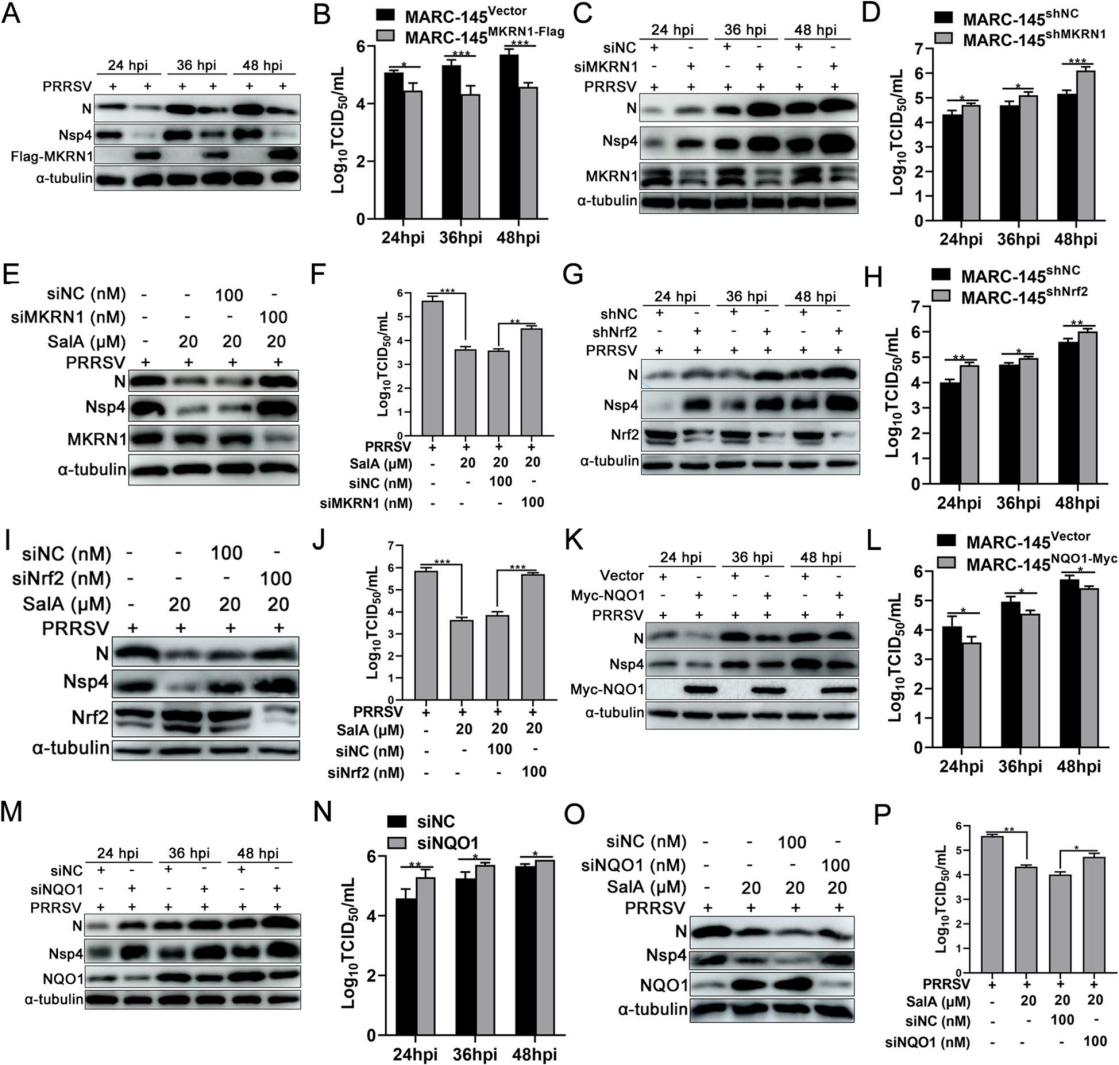
**SalA inhibits PRRSV replication by activating the MKRN1/Nrf2/NQO1 pathway.** **A** and **B** Effect of MKRN1 overexpression on PRRSV replication in MARC-145 cells. **C** and **D** Effect of MKRN1 knockdown on PRRSV replication in MARC-145 cells. **E** and **F** Effect of MKRN1 knockdown on SalA-mediated inhibition of PRRSV replication. **G** and **H** Effects of Nrf2 knockdown on PRRSV replication in MARC-145 cells. **I** and **J** Effect of Nrf2 knockdown on SalA-mediated suppression of PRRSV replication in MARC-145 cells. **K** and **L** Effects of NQO1 overexpression on PRRSV replication in MARC-145 cells. **M** and **N** Effects of NQO1 knockdown on PRRSV replication in MARC-145 cells. **O** and **P** Effect of NQO1 knockdown on the SalA-mediated inhibitory effect on PRRSV replication in MARC-145 cells.

Next, whether SalA exerts anti-PRRSV activity via the MKRN1/Nrf2/NQO1 pathway was investigated. The overexpression of MKRN1 in MARC-145 cells suppressed PRRSV N and Nsp4 protein expression at 24, 36, and 48 hpi (Figure 7A). TCID_50_ analysis of the supernatant viral titres suggested that, compared with the control, the overexpression of MKRN1 decreased the supernatant viral titre by 0.63 log_10_, 1.0 log_10_, and 1.12 log_10_, respectively (Figure 7B). Consistent with the above results, the knockdown of MKRN1 increased PRRSV N and Nsp4 protein expression (Figure 7C) and increased supernatant viral titres (0.39 log_10_,0.41 log_10_, and 0.95 log_10_) compared with those in the corresponding control groups (Figure 7D). Additionally, knockdown of MKRN1 partially reversed the inhibitory effect of SalA on PRRSV, as demonstrated by western blot analysis of the PRRSV N and Nsp4 proteins (Figure 7E) and TCID_50_ analysis of viral titres (Figure 7F).

Compared with that in the control group, Nrf2 knockdown in MARC-145 cells increased the protein expression of PRRSV N and Nsp4 at 24, 36, and 48 hpi (Figure 7G), and the supernatant progeny viral titre increased by 0.67 log_10_, 0.25 log_10_, and 0.41 log_10_, respectively (Figure 7H). Furthermore, Nrf2 knockdown partially reversed the suppressive effect of SalA on PRRSV replication, as shown by western blot analysis and TCID_50_ assay results (Figures 7I and 7J). The overexpression of NQO1 downregulated the protein expression of PRRSV N and Nsp4 in MARC-145 cells at 24, 36, and 48 hpi (Figure 7K). TCID_50_ analysis revealed that the titres were reduced by 0.55 log_10_, 0.41 log_10_, and 0.3 log_10_, respectively (Figure 7L). In contrast, the knockdown of NQO1 led to the upregulation of PRRSV N and Nsp4 protein expression (Figure 7M), and the supernatant viral titres increased by 0.71 log_10_, 0.45 log_10_, and 0.21 log_10_, respectively (Figure 7N). Moreover, knockdown of NQO1 partially reversed the inhibitory effect of SalA on PRRSV replication, as shown by western blot analysis and TCID_50_ assay results (Figures 7O and 7P). In summary, these results suggest that SalA inhibits PRRSV replication by activating the MKRN1/Nrf2/NQO1 pathway.

### SalA effectively alleviates PRRSV- and LPS-induced inflammatory responses by activating the Nrf2-mediated pathway

**Figure 8:**
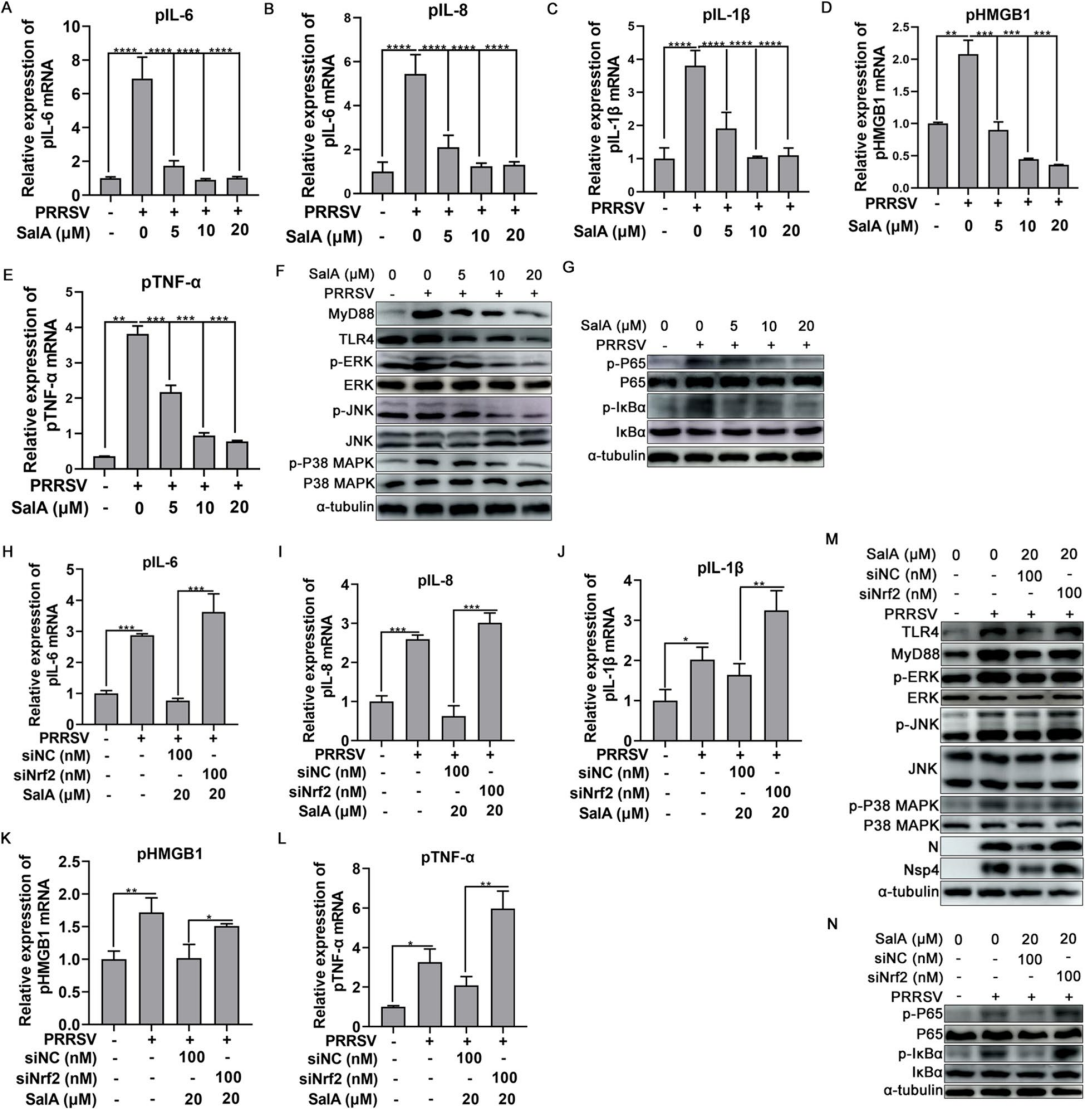
**SalA inhibits PRRSV-induced inflammatory cytokine and signalling pathway activation by activating the transcription factor Nrf2.** PAMs were infected with 0.01 MOI of PRRSV. At 1 hpi, the cells were treated with 0, 5, 10, or 20 μM SalA. At 24 hpi, the cells were collected for detection of the expression of the indicated cytokines or proteins. qRT-PCR detection of the effects of SalA on the PRRSV-induced upregulation of *pIL-6* (**A**), *pIL-8* (**B**), *pIL-1β* (**C**), *pHMGB1* (**D**) and *pTNF-α* (**E**) expression. **F** Western blot analysis of the effect of SalA on PRRSV-induced TLR4/MyD88-MAPK pathway activation. **G** Western blot analysis of the effect of SalA on PRRSV-induced NF-κB pathway activation. **H**–**L**) qRT-PCR detection of the effects of Nrf2 knockdown on SalA-mediated inhibition of PRRSV-induced upregulation of *pIL-6* (**H**), *pIL-8* (**I**), *pIL-1β* (**J**), *pHMGB1* (**K**), and *pTNF-α* (**L**) expression. **M** Effect of Nrf2 knockdown on SalA-mediated inhibition of the TLR4/MyD88-MAPK pathway. **N** Effect of Nrf2 knockdown on SalA-mediated inhibition of the NF-κB pathway.

PRRSV infection induces high levels of inflammatory cytokines, which are associated with acute lung injury (ALI) [49, 50]. These results confirmed that SalA treatment activated downstream signalling pathways centered on Nrf2. Thus, whether SalA is involved in regulating the PRRSV-induced inflammatory response was determined. PRRSV infection of PAMs dramatically induced the expression of inflammation-related cytokines, such as porcine *IL-6 (pIL-6)*, porcine *IL-8 (pIL-8),* porcine *IL-1β (pIL-1β)*, porcine *HMGB1 (pHMGB1)*, and porcine *TNF-α (pTNF-α)* (Figures 8A–E). However, compared with PRRSV infection, SalA treatment dose-dependently repressed the mRNA expression of these inflammatory cytokines (Figures 8A–E). The TLR4/MyD88-MAPK and TLR4/MyD88-NF-κB signalling pathways mediate the inflammatory response and are associated with inflammation-related ALI caused by PRRSV [49, 51]. Therefore, whether SalA affects the TLR4/MyD88-MAPK and TLR4/MyD88-NF-κB signalling pathways during PRRSV infection was determined. The results indicated that PRRSV infection indeed promoted the expression of TLR4, MyD88, p-P38-MAPK, p-JNK, and p-ERK in PAMs (Fig. 8F). Additionally, PRRSV infection activated the NF-κB pathway, as evidenced by the upregulation of p-P65 and p-IκBα protein expression (Figure 8G). Nevertheless, SalA treatment suppressed the PRRSV-induced upregulation of the TLR4, MyD88, p-P38-MAPK, p-JNK, and p-ERK proteins without affecting the total P38-MAPK, JNK, or ERK levels (Figure 8F). SalA treatment also suppressed the PRRSV-induced upregulation of p-P65 and p-IκBα but not total P65 or total IκBα (Figure 8G). Considering that TLR4 and MyD88 simultaneously participate in the activation of downstream MAPK and NF-κB pathways, these results suggest that SalA effectively alleviates the inflammatory response during PRRSV infection by suppressing the TLR4/MyD88-MAPK and TLR4/MyD88-NF-κB signalling pathways.

Next, whether Nrf2 mediates the anti-inflammatory effect of SalA was further determined. PAMs were first infected with PRRSV, treated with various concentrations of SalA, and finally transfected with siNC or siNrf2. The qRT-PCR results revealed that SalA effectively reduced the PRRSV-induced expression of *pIL-6*, *pIL-8*, *pIL-1β*, *pHMGB1*, and *pTNF-α*; however, Nrf2 knockdown effectively reversed the inhibitory effect of SalA on inflammatory gene expression (Figures 8H‒L). Whether Nrf2 mediated SalA-mediated inhibition of TLR4/MyD88-MAPK and TLR4/MyD88-NF-κB pathway activation during PRRSV infection was also determined. As shown in Figure 8M, SalA treatment inhibited PRRSV-induced TLR4, MyD88, p-P38-MAPK, p-JNK, and p-ERK protein expression in PAMs, which was partially reversed by Nrf2 knockdown. In addition, Nrf2 knockdown partially reversed the SalA-mediated reduction in p-P65 and p-IκBα expression without affecting the expression of P65 and IκBα (Figure 8N). These results suggest that the transcription factor Nrf2 acts as a key switch in SalA-mediated inhibition of the inflammatory response during PRRSV infection.

PRRSV infection is often accompanied by secondary bacterial infection, which ultimately leads to the release of abundant LPS into the pig’s body and causes inflammatory injury. Thus, the effects of SalA on LPS-induced inflammatory cytokines and the activation of related signalling pathways were further detected. LPS obviously induced *pIL-6*, *pIL-8*, *pIL-1β*, *pHMGB1*, and *pTNF-α* expression in PAMs (Additional files 2A–E); however, SalA treatment distinctly inhibited LPS-induced inflammatory cytokine expression, and knockdown of Nrf2 partially reversed the inhibitory effect of SalA on the expression of these inflammatory factors (Additional files 2H, L). Further studies revealed that SalA treatment suppressed LPS-induced TLR4/MyD88-MAPK and TLR4/MyD88-NF-κB pathway activation, as indicated by the reduced expression of the TLR4, MyD88, p-P38-MAPK, p-JNK, p-ERK, p-P65, and p-IκBα proteins (Additional files 2F and G), whereas Nrf2 knockdown partially abolished the suppressive effect of SalA (Additional files 2 M and N). These results suggest that SalA inhibits the LPS-induced upregulation of inflammatory cytokines and the activation of related signalling pathways by activating Nrf2.

### SalA suppresses PRRSV- and LPS-induced inflammasome activation and pyroptosis by activating Nrf2

**Figure 9:**
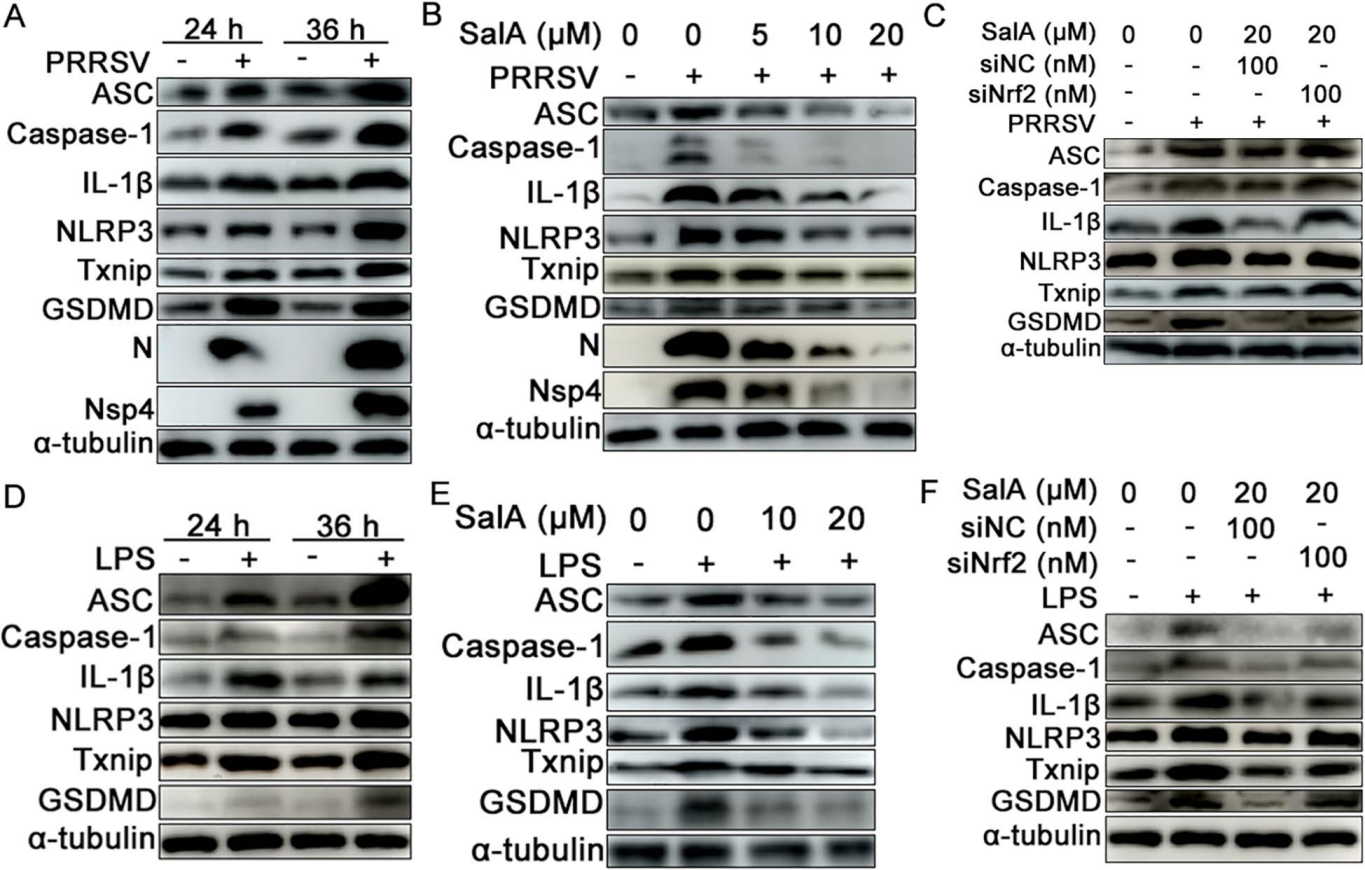
**SalA inhibits the activation of inflammasome- and pyroptosis-related signalling pathways by activating the transcription factor Nrf2.** **A** PAMs were infected with 0.01 MOI PRRSV. At 24 and 36 hpi, the cells were harvested to detect the expression of ASC, caspase-1, IL-1β, NLRP3, Txnip, and GSDMD via western blotting. **B** Effects of SalA on PRRSV-activated ASC, caspase-1, IL-1β, NLRP3, Txnip, and GSDMD expression. **C** Effects of Nrf2 knockdown on SalA-mediated inhibition of ASC, caspase-1, IL-1β, NLRP3, Txnip, and GSDMD expression. **D** PAMs were treated with LPS (1 mg/mL), and the cells were harvested at 24 and 36 h. The expression of ASC, caspase-1, IL-1β, NLRP3, Txnip, and GSDMD was detected by western blotting. **E** Effects of SalA on the LPS-induced activation of ASC, caspase-1, IL-1β, NLRP3, Txnip, and GSDMD expression.

Previous studies have shown that PRRSV infection activates the NLRP3 inflammasome and GSDMD-mediated cellular pyroptosis, which are associated with microscopic porcine lung lesions [9]. To detect the expression of inflammasome- and pyroptosis-related proteins, PAMs were infected with PRRSV. As shown in Figure 9A, PRRSV infection increased the expression of ASC, caspase-1, IL-1β, NLRP3, Txnip, and GSDMD compared with that in uninfected control PAMs, which was consistent with the findings of previous studies [9]. However, SalA treatment concentration-dependently suppressed the expression of the proteins ASC, caspase-1, IL-1β, NLRP3, Txnip, and GSDMD induced by PRRSV in a concentration-dependent manner (Figure 9B). Whether Nrf2 plays a pivotal role in SalA-mediated inhibition of NLRP3 inflammasome activation and pyroptosis was also investigated. As shown in Figure 9C, SalA treatment suppressed the protein expression of PRRSV-induced ASC, caspase-1, IL-1β, NLRP3, Txnip, and GSDMD. However, Nrf2 knockdown partially abolished the suppressive effect of SalA on the expression of NLRP3 inflammasome- and pyroptosis-related proteins during PRRSV infection (Figure 9C).

The effects of SalA on LPS-induced inflammasome and pyroptosis activation were subsequently detected. The results indicated that LPS treatment upregulated the expression of NLRP3 inflammasome-related proteins (ASC, caspase-1, IL-1β, NLRP3, and Txnip) and the pyroptosis-related protein GSDMD (Figure 9D). Nevertheless, SalA inhibited the expression of LPS-induced NLRP3 inflammasome- and pyroptosis-related proteins (Figure 9E). Knockdown of Nrf2 partially abolished the suppressive effect of SalA on the expression of LPS-induced NLRP3 inflammasome- and pyroptosis-related proteins (Figure 9F). These results suggest that the transcription factor Nrf2 plays a pivotal role in mediating the ability of SalA to suppress NLRP3 inflammasome activation and cellular pyroptosis during PRRSV infection and secondary bacterial infections.

## Discussion

Despite extensive and in-depth research on PRRS and its causative agent, PRRSV, the prevention and control of PRRS remain major challenges for the global pig industry [52, 53]. Owing to the continuous emergence of new strains and complex immune evasion mechanisms, existing commercial vaccines often fail to provide comprehensive and effective protective immunity. In addition to vaccines, the discovery and development of antiviral medications are pivotal for the prevention and control of animal diseases. In this study, the role and mechanisms of the traditional Chinese medicinal component SalA in PRRSV replication were explored. SalA significantly suppressed PRRSV replication in vitro and in vivo, and its antiviral activity largely depended on the activation of the MKRN1/Nrf2/NQO1 pathway. Furthermore, SalA effectively alleviated PRRSV-induced expression of inflammatory cytokines, inhibited inflammasome activation, and reduced pyroptosis. SalA directly binds to the Thr560 residue of Keap1, promoting the recruitment of the E3 ligase MKRN1 to facilitate the proteasomal degradation of Keap1; this, in turn, promotes the nuclear translocation of Nrf2 and the subsequent activation of antiviral and anti-inflammatory pathways.

The potential mechanism by which SalA regulates the Keap1/Nrf2 pathway was investigated. SalA directly binds to the Thr560 site of Keap1 via hydrogen bonds to promote the recruitment of a newly identified E3 ubiquitin ligase, MKRN1, leading to K48-linked ubiquitination of Keap1 at the K615 residue, subsequent proteasomal degradation, and eventual activation of the Nrf2–NQO1 pathway. Under physiological conditions, Nrf2 is sequestered in the cytoplasm by its inhibitor Keap1, where it is constantly degraded via the ubiquitin‒proteasome pathway [54, 55]. As SalA treatment degraded Keap1 and promoted Nrf2 expression, it was hypothesized that Keap1 was degraded via the ubiquitin‒proteasome pathway. These results confirmed that SalA promoted the recruitment of the E3 ligase MKRN1 by Keap1, leading to K48-linked ubiquitination of Keap1 and subsequent degradation via the ubiquitin‒proteasome pathway. MKRN1 belongs to the makorin RING finger protein family, the members of which possess E3 ubiquitin ligase activity [56]. The present study was the first to demonstrate its interaction with Keap1 and its relationship with the Nrf2-mediated anti-inflammatory response. Specifically, SalA may serve as a molecular glue to promote Keap1 degradation by recruiting the E3 ligase MKRN1, a mechanism that has not been reported previously. Therefore, SalA might provide a novel molecular template for the development of anti-PRRSV drug candidates by activating the function of the ubiquitin–proteasome system. Studies have shown that SalA can effectively target the structural protein gB of herpes simplex virus and inhibit viral replication [57]. In addition, SalA can directly bind to the 3C protease of enterovirus 71, exhibiting potent antiviral activity [58]. SalA can also bind to both the receptor-binding domain (RBD) of the SARS-CoV-2 spike protein and the cell surface receptor ACE2, thereby inhibiting viral replication [59]. The effects of another Nrf2 inducer, sulforaphane, on PRRSV replication and the activity of the Keap1‒Nrf2 pathway were also analysed. Although sulforaphane can also activate the Keap1‒Nrf2 pathway and inhibit PRRSV replication (Additional file 3), its mechanism of activating the Keap1‒Nrf2 pathway is distinct from that of SalA. Specifically, sulforaphane did not promote Keap1 ubiquitination, and its binding sites on Keap1 were also different from those of SalA (Additional file 3). In contrast, our results confirmed that SalA directly bound to the host Keap1 protein to promote its recruitment of MKRN1, induce Keap1 degradation, and then activate the Nrf2‒NQO1 pathway to inhibit PRRSV replication. These studies indicate that SalA inhibits viral replication through different mechanisms for different viruses, either by targeting key viral proteins or by activating the host’s antiviral defense response. Considering the ancient origin and high conservation of the Keap1‒Nrf2 pathway in exerting antioxidative stress-protective functions in mammals, the antiviral activity of SalA—achieved by degrading Keap1 to activate the Nrf2 pathway—should involve broad-spectrum antiviral activity rather than PRRSV-specific activity. Notably, whether SalA directly targets specific PRRSV proteins to block viral replication remains unclear and warrants further investigation. However, this study demonstrated that activating Nrf2-related pathways is one of the key mechanisms by which SalA exerts its antiviral activity.

PRRSV infection is always accompanied by severe interstitial pneumonia and pathological damage, suggesting that the inflammatory response plays an important role in PRRSV pathogenesis [49, 60]. The present results indicated that PRRSV infection markedly upregulated the expression of proinflammatory cytokines, such as *pIL-1β*, *pIL-6*, and *pTNF-α* (Figure 8). However, treatment with SalA reversed this upregulation of proinflammatory cytokines, demonstrating that SalA exerts a significant anti-inflammatory effect during PRRSV infection. An increasing number of studies suggest that the synergistic effects of PRRSV and secondary bacterial infection can induce a more severe inflammatory response than single PRRSV infection or single bacterial infection [18, 61–63]. The present study revealed that, in addition to PRRSV treatment, SalA treatment inhibited the LPS-induced inflammatory response, which is consistent with previous studies reporting the anti-inflammatory activity of SalA [28]. Previous studies have demonstrated that PRRSV induces the production of inflammatory cytokines in a manner dependent on the TLR4/MyD88/NF-κB pathway and NLRP3 inflammasome activation [51, 64, 65].

TLR4 recognizes extracellular LPS and triggers the activation of the TLR4‒MyD88 pathway, followed by the activation of NF-κB via the phosphorylation and subsequent degradation of IκBα, as well as the phosphorylation of P38, ERK, and JNK. This process leads to the production of inflammatory factors and ultimately causes lung inflammation [66]. SalA inhibited p65 phosphorylation, blocked PRRSV- and LPS-induced activation of TLR4 and MyD88, and inhibited the degradation of IκBα in PRRSV-infected or LPS-treated PAMs. Furthermore, SalA effectively reduced the expression of PRRSV- and LPS-induced p-P38 MAPK, p-JNK, and p-ERK. These findings indicate that SalA attenuates PRRSV-induced inflammatory responses, in part, by inactivating the TLR4/MyD88-MAPK and TLR4/MyD88-NF-κB signalling pathways.

The NLRP3 inflammasome complex is composed of NLRP3, ASC, and caspase-1. Its activation is characterized by the activation of caspase-1 and the cleavage of pro-IL-1β to form mature IL-1β; this activation process is closely associated with inflammatory responses [65]. The activation of NF-κB is an essential initial step for the priming of NLRP3 activation, and ROS derived from NF-κB-mediated inflammation also participate in NLRP3 activation [25]. The results of the present study revealed that SalA inhibited PRRSV- and LPS-induced expression of NLRP3, caspase-1, and ASC. Nrf2 plays a central role in mediating the antiviral and anti-inflammatory effects of SalA, as knockdown of Nrf2 partially reversed the inhibitory effects of SalA on PRRSV replication and inflammatory responses. Additionally, knockdown of Nrf2 partially reversed the inhibitory effect of SalA on the TLR4/MyD88-MAPK and TLR4/MyD88-NF-κB pathways, emphasizing the core role of Nrf2 in the protective biological functions of SalA. However, how Nrf2 regulates the TLR4/MyD88-MAPK and -NF-κB pathways requires further investigation. Our previous studies revealed that PRRSV infection induced the accumulation of intracellular ROS [67]. Although the present study did not investigate the antioxidant effect of SalA or the role of ROS in pyroptosis, it is reasonable to speculate that SalA might exert an antioxidant effect to prevent ROS-induced pyroptosis, and this requires further investigation.

## Supplementary Information

**Additional file 1: IRF2BP1 or MKRN3 did not catalyze the ubiquitination of Keap1**. (A) Verification of the expression of recombinant Flag-MKRN3. (B) HEK293T cells were cotransfected with Flag-MKRN3 or Myc-Keap1 together with HA-Ub for 48 h, after which the cells were harvested for analysis of Keap1 ubiquitination usin a co-IP assay. (C) Verification of the expression of recombinant GFP-IRF2BP1. (D) Effect of IRF2BP1 overexpression on the ubiquitination of Keap1.

**Additional file 2: SalA inhibits LPS-induced inflammation- and pyroptosis-related signalling pathways by activating the transcription factor Nrf2. PAMs were treated with LPS (1 mg/mL) for 12 h and then treated with 0, 5, 10, or 20 μM SalA**. After treatment for 24 h, the cells were harvested. The expression of *pIL-6* (A), *pIL-8* (B), *pIL-1β* (C), *pHMGB1* (D) and *pTNF-α* (E) was detected by qRT-PCR. (F) Effect of SalA on LPS-induced TLR4/MyD88-MAPK pathway activation. (G) Effect of SalA on LPS-induced NF-κB pathway activation. Effect of Nrf2 knockdown on the inhibitory effects of SalA on *pIL-6* (H), *pIL-8* (I), *pIL-1β* (J), *pHMGB1* (K) and *pTNF-α* (L) levels. (O) Effect of Nrf2 knockdown on SalA-mediated inhibition of the TLR4/MyD88-MAPK pathway. (P) Effect of Nrf2 knockdown on SalA-mediated inhibition of the NF-κB pathway.

**Additional file 3: Effects of sulforaphane on PRRSV replication and the activity of the Keap1-Nrf2 pathway.** Effect of sulforaphane on PRRSV replication in MARC-145 cells: (A) expression of the PRRSV N protein; (B) supernatant progeny viral titres. (C) Effects of sulforaphane on Keap1-Nrf2 pathway activity. (D) Docked conformation of sulforaphane with Keap1. (E) Effect of sulforaphane on the ubiquitination of Keap1.

## Data Availability

The datasets used during and/or analysed during the current study are available from the corresponding author upon reasonable request.

## References

[1] Meulenberg JJ (2000) PRRSV, the virus. Vet Res 31:11–21 10726635 10.1051/vetres:2000103

[2] Tong GZ, Zhou YJ, Hao XF, Tian ZJ, An TQ, Qiu HJ (2007) Highly pathogenic porcine reproductive and respiratory syndrome, China. Emerg Infect Dis 13:1434–1436 18252136 10.3201/eid1309.070399 PMC2857295

[3] Wu Y, Lin L, Gao X, Zheng J, Yin L, Zhao H, Ren B, Wang L, Li Q (2024) Evaluation of the cross-protective effect of VR2332 modified live virus vaccine against a recombinant NADC34-like porcine reproductive and respiratory syndrome virus. Front Vet Sci 11:1472960 39641098 10.3389/fvets.2024.1472960 PMC11618057

[4] Lunney JK, Fang Y, Ladinig A, Chen N, Li Y, Rowland B, Renukaradhya GJ (2016) Porcine reproductive and respiratory syndrome virus (PRRSV): pathogenesis and Interaction with the Immune System. Annu Rev Anim Biosci 4:129–154 26646630 10.1146/annurev-animal-022114-111025

[5] Pejsak Z, Stadejek T, Markowska-Daniel I (1997) Clinical signs and economic losses caused by porcine reproductive and respiratory syndrome virus in a large breeding farm. Vet Microbiol 55:317–322 9220628 10.1016/s0378-1135(96)01326-0

[6] Chen N, Li X, Xiao Y, Li S, Zhu J (2021) Characterization of four types of MLV-derived porcine reproductive and respiratory syndrome viruses isolated in unvaccinated pigs from 2016 to 2020. Res Vet Sci 134:102–111 33360570 10.1016/j.rvsc.2020.12.007

[7] Yuan L, Zhu Z, Fan J, Liu P, Li Y, Li Q, Sun Z, Yu X, Lee HS, Tian K, Li X (2022) High pathogenicity of a Chinese NADC34-like PRRSV on pigs. Microbiol Spectr 10:e0154122 35766496 10.1128/spectrum.01541-22 PMC9431460

[8] Nelsen CJ, Murtaugh MP, Faaberg KS (1999) Porcine reproductive and respiratory syndrome virus comparison: divergent evolution on two continents. J Virol 73:270–280 9847330 10.1128/jvi.73.1.270-280.1999 PMC103831

[9] He S, Li L, Chen H, Hu X, Wang W, Zhang H, Wei R, Zhang X, Chen Y, Liu X (2022) PRRSV infection induces gasdermin D-driven pyroptosis of porcine alveolar macrophages through NLRP3 inflammasome activation. J Virol 96:e0212721 35758658 10.1128/jvi.02127-21 PMC9327688

[10] Chen D, Xu S, Jiang R, Guo Y, Yang X, Zhang Y, Zhou L, Ge X, Han J, Guo X, Yang H (2022) IL-1beta induced by PRRSV co-infection inhibited CSFV C-strain proliferation via the TLR4/NF-kappaB/MAPK pathways and the NLRP3 inflammasome. Vet Microbiol 273:109513 35952491 10.1016/j.vetmic.2022.109513

[11] Xu Y, Wang H, Zhang X, Zheng X, Zhu Y, Han H, Feng WH (2021) Highly pathogenic porcine reproductive and respiratory syndrome virus (HP-PRRSV) induces IL-6 production through TAK-1/JNK/AP-1 and TAK-1/NF-kappaB signaling pathways. Vet Microbiol 256:109061 33836390 10.1016/j.vetmic.2021.109061

[12] Liu Y, Du Y, Wang H, Du L, Feng WH (2017) Porcine reproductive and respiratory syndrome virus (PRRSV) up-regulates IL-8 expression through TAK-1/JNK/AP-1 pathways. Virology 506:64–72 28347884 10.1016/j.virol.2017.03.009 PMC7111726

[13] Gong X, Ma T, Wang J, Cao X, Zhang Q, Wang Y, Song C, Lai M, Zhang C, Fang X, Chen X (2023) Nucleocapsid protein residues 35, 36, and 113 are critical sites in up-regulating the interleukin-8 production via C/EBPalpha pathway by highly pathogenic porcine reproductive and respiratory syndrome virus. Microb Pathog 184:106345 37714310 10.1016/j.micpath.2023.106345

[14] He Q, Li Y, Zhou L, Ge X, Guo X, Yang H (2015) Both Nsp1beta and Nsp11 are responsible for differential TNF-alpha production induced by porcine reproductive and respiratory syndrome virus strains with different pathogenicity *in vitro*. Virus Res 201:32–40 25708177 10.1016/j.virusres.2015.02.014

[15] Mangan MSJ, Olhava EJ, Roush WR, Seidel HM, Glick GD, Latz E (2018) Targeting the NLRP3 inflammasome in inflammatory diseases. Nat Rev Drug Discov 17:688 30116046 10.1038/nrd.2018.149

[16] XiangjinYan ZJ, Li X, Zhang Z, Din AU, Zhao K, Zhou Y (2020) High incidence and characteristic of PRRSV and resistant bacterial Co-Infection in pig farms. Microb Pathog 149:104536 32980472 10.1016/j.micpath.2020.104536

[17] Zhang J, Wang J, Zhang X, Zhao C, Zhou S, Du C, Tan Y, Zhang Y, Shi K (2022) Transcriptome profiling identifies immune response genes against porcine reproductive and respiratory syndrome virus and *Haemophilus parasuis* co-infection in the lungs of piglets. J Vet Sci 23:e2 34931503 10.4142/jvs.21139 PMC8799943

[18] Yao X, Dai W, Yang S, Wang Z, Zhang Q, Meng Q, Zhang T (2023) Synergistic effect of treatment with highly pathogenic porcine reproductive and respiratory syndrome virus and lipopolysaccharide on the inflammatory response of porcine pulmonary microvascular endothelial cells. Viruses 15:1523 37515210 10.3390/v15071523 PMC10383901

[19] Labarque G, Van Reeth K, Van Gucht S, Nauwynck H, Pensaert M (2002) Porcine reproductive-respiratory syndrome virus infection predisposes pigs for respiratory signs upon exposure to bacterial lipopolysaccharide. Vet Microbiol 88:1–12 12119134 10.1016/S0378-1135(02)00104-9 PMC7117251

[20] Kuzmich NN, Sivak KV, Chubarev VN, Porozov YB, Savateeva-Lyubimova TN, Peri F (2017) TLR4 signaling pathway modulators as potential therapeutics in inflammation and sepsis. Vaccines (Basel) 5:34 28976923 10.3390/vaccines5040034 PMC5748601

[21] Chang WT, Bow YD, Fu PJ, Li CY, Wu CY, Chang YH, Teng YN, Li RN, Lu MC, Liu YC, Chiu CC (2021) A marine terpenoid, heteronemin, induces both the apoptosis and ferroptosis of hepatocellular carcinoma cells and involves the ROS and MAPK pathways. Oxid Med Cell Longev 2021:7689045 33488943 10.1155/2021/7689045 PMC7803406

[22] Lee YJ, Lee C (2012) Stress-activated protein kinases are involved in porcine reproductive and respiratory syndrome virus infection and modulate virus-induced cytokine production. Virology 427:80–89 22424736 10.1016/j.virol.2012.02.017

[23] Yu J, Liu Y, Zhang Y, Zhu X, Ren S, Guo L, Liu X, Sun W, Chen Z, Cong X, Chen L, Shi J, Du Y, Li J, Wu J, Wang J (2017) The integrity of PRRSV nucleocapsid protein is necessary for up-regulation of optimal interleukin-10 through NF-kappaB and p38 MAPK pathways in porcine alveolar macrophages. Microb Pathog 109:319–324 28457899 10.1016/j.micpath.2017.04.036

[24] Chen J, Chen ZJ (2018) PtdIns4P on dispersed trans-Golgi network mediates NLRP3 inflammasome activation. Nature 564:71–76 30487600 10.1038/s41586-018-0761-3 PMC9402428

[25] Bauernfeind FG, Horvath G, Stutz A, Alnemri ES, MacDonald K, Speert D, Fernandes-Alnemri T, Wu J, Monks BG, Fitzgerald KA, Hornung V, Latz E (2009) Cutting edge: NF-kappaB activating pattern recognition and cytokine receptors license NLRP3 inflammasome activation by regulating NLRP3 expression. J Immunol 183:787–791 19570822 10.4049/jimmunol.0901363 PMC2824855

[26] Buscetta M, Di Vincenzo S, Miele M, Badami E, Pace E, Cipollina C (2020) Cigarette smoke inhibits the NLRP3 inflammasome and leads to caspase-1 activation via the TLR4-TRIF-caspase-8 axis in human macrophages. FASEB J 34:1819–1832 31914643 10.1096/fj.201901239R

[27] Kang TH, Shin S, Park J, Lee BR, Lee SI (2023) Pyroptosis-mediated damage mechanism by deoxynivalenol in porcine small intestinal epithelial cells. Toxins (Basel) 15:300 37104238 10.3390/toxins15040300 PMC10146237

[28] Zhang HF, Wang YL, Gao C, Gu YT, Huang J, Wang JH, Wang JH, Zhang Z (2018) Salvianolic acid A attenuates kidney injury and inflammation by inhibiting NF-kappaB and p38 MAPK signaling pathways in 5/6 nephrectomized rats. Acta Pharmacol Sin 39:1855–1864 29795135 10.1038/s41401-018-0026-6 PMC6289371

[29] Martins-Gomes C, Nunes FM, Silva AM (2024) *Thymus* spp. aqueous extracts and their constituent salvianolic acid A induce Nrf2-dependent cellular antioxidant protection against oxidative stress in Caco-2 cells. Antioxidants (Basel) 13:1287 39594429 10.3390/antiox13111287 PMC11591053

[30] Xie D, Song L, Xiang D, Gao X, Zhao W (2023) Salvianolic acid A alleviates atherosclerosis by inhibiting inflammation through Trc8-mediated 3-hydroxy-3-methylglutaryl-coenzyme A reductase degradation. Phytomedicine 112:154694 36804757 10.1016/j.phymed.2023.154694

[31] Fan X, Zhang L, La X, Tian J, Israr G, Li A, Wu C, An Y, Li S, Dong X, Li Z (2023) Salvianolic acid A attenuates inflammation-mediated atherosclerosis by suppressing GRP78 secretion of endothelial cells. J Ethnopharmacol 308:116219 36758912 10.1016/j.jep.2023.116219

[32] Chen Z, Li D, Wang T, Li Y, Qin P, Zhu H, Zhang M, Li W, Yu L, Duan H, Chen L, Li Y, Zheng G (2024) Salvianolic acid A inhibits pseudorabies virus infection by directly inactivating the virus particle. Phytomedicine 134:156015 39244942 10.1016/j.phymed.2024.156015

[33] Hu S, Wang J, Zhang Y, Bai H, Wang C, Wang N, He L (2021) Three salvianolic acids inhibit 2019-nCoV spike pseudovirus viropexis by binding to both its RBD and receptor ACE2. J Med Virol 93:3143–3151 33580518 10.1002/jmv.26874 PMC8013543

[34] Liu X, Bai J, Jiang C, Song Z, Zhao Y, Nauwynck H, Jiang P (2019) Therapeutic effect of Xanthohumol against highly pathogenic porcine reproductive and respiratory syndrome viruses. Vet Microbiol 238:108431 31648725 10.1016/j.vetmic.2019.108431

[35] Song Z, Bai J, Nauwynck H, Lin L, Liu X, Yu J, Jiang P (2019) 25-hydroxycholesterol provides antiviral protection against highly pathogenic porcine reproductive and respiratory syndrome virus in swine. Vet Microbiol 231:63–70 30955825 10.1016/j.vetmic.2019.02.035

[36] Duan H, Chen X, Zhang Z, Zhang Z, Li Z, Wang X, Zhao J, Nan Y, Liu B, Zhang A, Sun Y, Zhao Q (2024) A nanobody inhibiting porcine reproductive and respiratory syndrome virus replication via blocking self-interaction of viral nucleocapsid protein. J Virol 98:e0131923 38084961 10.1128/jvi.01319-23 PMC10804987

[37] National Library of Medicine. https://pubchem.ncbi.nlm.nih.gov/compound/5281793. Accessed 20 Jan 2025

[38] AlphaFold Protein Structure Database. https://alphafold.ebi.ac.uk/. Accessed 12 Jan 2025

[39] Gaillard T (2018) Evaluation of AutoDock and AutoDock Vina on the CASF-2013 benchmark. J Chem Inf Model 58:1697–1706 29989806 10.1021/acs.jcim.8b00312

[40] Baird L, Dinkova-Kostova AT (2011) The cytoprotective role of the Keap1-Nrf2 pathway. Arch Toxicol 85:241–272 21365312 10.1007/s00204-011-0674-5

[41] Ross D, Siegel D (2021) The diverse functionality of NQO1 and its roles in redox control. Redox Biol 41:101950 33774477 10.1016/j.redox.2021.101950 PMC8027776

[42] Ahmed SM, Luo L, Namani A, Wang XJ, Tang X (2017) Nrf2 signaling pathway: Pivotal roles in inflammation. Biochim Biophys Acta Mol Basis Dis 1863:585–597 27825853 10.1016/j.bbadis.2016.11.005

[43] Zhu Z, Luo Y, Lou G, Yihunie K, Wizzard S, DeVilbiss AW, Muh S, Ma C, Shinde SS, Hoar J, Hu T, Zhang N, Biswal S, DeBerardinis RJ, Wu T, Yao C (2024) The redox sensor KEAP1 facilitates adaptation of T cells to chronic antigen stimulation by preventing hyperactivation. Sci Immunol 9:eadk2954 39612322 10.1126/sciimmunol.adk2954 PMC12155420

[44] Erpapazoglou Z, Walker O, Haguenauer-Tsapis R (2014) Versatile roles of k63-linked ubiquitin chains in trafficking. Cells 3:1027–1088 25396681 10.3390/cells3041027 PMC4276913

[45] Tan JM, Wong ES, Kirkpatrick DS, Pletnikova O, Ko HS, Tay SP, Ho MW, Troncoso J, Gygi SP, Lee MK, Dawson VL, Dawson TM, Lim KL (2008) Lysine 63-linked ubiquitination promotes the formation and autophagic clearance of protein inclusions associated with neurodegenerative diseases. Hum Mol Genet 17:431–439 17981811 10.1093/hmg/ddm320

[46] Matsumoto G, Wada K, Okuno M, Kurosawa M, Nukina N (2011) Serine 403 phosphorylation of p62/SQSTM1 regulates selective autophagic clearance of ubiquitinated proteins. Mol Cell 44:279–289 22017874 10.1016/j.molcel.2011.07.039

[47] Prediction of SUMOylation Sites & SUMO-binding Motifs. http://gpsuber.biocuckoo.cn/online.php. Accessed 20 Nov 2024

[48] UbiBrowser 2.0. http://ubibrowser.bio-it.cn/ubibrowser_v3/Home/Result/index/name/Q684M4/module/Strict/proteinType/noE3. Accessed 20 Dec 2024

[49] Han D, Hu Y, Li L, Tian H, Chen Z, Wang L, Ma H, Yang H, Teng K (2014) Highly pathogenic porcine reproductive and respiratory syndrome virus infection results in acute lung injury of the infected pigs. Vet Microbiol 169:135–146 24472226 10.1016/j.vetmic.2013.12.022 PMC7127595

[50] Renson P, Rose N, Le Dimna M, Mahe S, Keranflec’h A, Paboeuf F, Belloc C, Le Potier MF, Bourry O (2017) Dynamic changes in bronchoalveolar macrophages and cytokines during infection of pigs with a highly or low pathogenic genotype 1 PRRSV strain. Vet Res 48:15 28241868 10.1186/s13567-017-0420-y PMC5327547

[51] Bi J, Song S, Fang L, Wang D, Jing H, Gao L, Cai Y, Luo R, Chen H, Xiao S (2014) Porcine reproductive and respiratory syndrome virus induces IL-1beta production depending on TLR4/MyD88 pathway and NLRP3 inflammasome in primary porcine alveolar macrophages. Mediators Inflamm 2014:403515 24966466 10.1155/2014/403515 PMC4055429

[52] Zhao HZ, Wang FX, Han XY, Guo H, Liu CY, Hou LN, Wang YX, Zheng H, Wang L, Wen YJ (2022) Recent advances in the study of NADC34-like porcine reproductive and respiratory syndrome virus in China. Front Microbiol 13:950402 35935186 10.3389/fmicb.2022.950402 PMC9354828

[53] Amadori M, Listorti V, Razzuoli E (2021) Reappraisal of PRRS immune control strategies: the way forward. Pathogens 10:1073 34578106 10.3390/pathogens10091073 PMC8469074

[54] Hayes JD, Dinkova-Kostova AT (2014) The Nrf2 regulatory network provides an interface between redox and intermediary metabolism. Trends Biochem Sci 39:199–218 24647116 10.1016/j.tibs.2014.02.002

[55] Suzuki T, Yamamoto M (2015) Molecular basis of the Keap1-Nrf2 system. Free Radic Biol Med 88:93–100 26117331 10.1016/j.freeradbiomed.2015.06.006

[56] Guseva EA, Emelianova MA, Sidorova VN, Tyulpakov AN, Dontsova OA, Sergiev PV (2024) Diversity of molecular functions of RNA-binding ubiquitin ligases from the MKRN protein family. Biochemistry (Mosc) 89:1558–1572 39418515 10.1134/S0006297924090037

[57] Yan H, Li H, Chen X, Wang J, Yang J, Xu Z, Hao C, Wang W (2025) Salvianolic acid A acts as a herpes simplex virus dual inhibitor by blocking glycoprotein B-mediated adsorption and membrane fusion. Phytomedicine 143:156910 40450985 10.1016/j.phymed.2025.156910

[58] Shi S, Xie L, Ma S, Xu B, An H, Ye S, Wang Y (2023) Computational and experimental studies of salvianolic acid A targets 3C protease to inhibit enterovirus 71 infection. Front Pharmacol 14:1118584 36937869 10.3389/fphar.2023.1118584 PMC10017496

[59] Zhang D, Hamdoun S, Chen R, Yang L, Ip CK, Qu Y, Li R, Jiang H, Yang Z, Chung SK, Liu L, Wong VKW (2021) Identification of natural compounds as SARS-CoV-2 entry inhibitors by molecular docking-based virtual screening with bio-layer interferometry. Pharmacol Res 172:105820 34403732 10.1016/j.phrs.2021.105820 PMC8364251

[60] Sun W, Wu W, Jiang N, Ge X, Zhang Y, Han J, Guo X, Zhou L, Yang H (2022) Highly pathogenic PRRSV-infected alveolar macrophages impair the function of pulmonary microvascular endothelial cells. Viruses 14:452 35336858 10.3390/v14030452 PMC8948932

[61] Van Gucht S, Labarque G, Van Reeth K (2004) The combination of PRRS virus and bacterial endotoxin as a model for multifactorial respiratory disease in pigs. Vet Immunol Immunopathol 102:165–178 15507303 10.1016/j.vetimm.2004.09.006 PMC7112634

[62] Qiao S, Feng L, Bao D, Guo J, Wan B, Xiao Z, Yang S, Zhang G (2011) Porcine reproductive and respiratory syndrome virus and bacterial endotoxin act in synergy to amplify the inflammatory response of infected macrophages. Vet Microbiol 149:213–220 21129861 10.1016/j.vetmic.2010.11.006

[63] Li J, Wang S, Li C, Wang C, Liu Y, Wang G, He X, Hu L, Liu Y, Cui M, Bi C, Shao Z, Wang X, Xiong T, Cai X, Huang L, Weng C (2017) Secondary *Haemophilus parasuis* infection enhances highly pathogenic porcine reproductive and respiratory syndrome virus (HP-PRRSV) infection-mediated inflammatory responses. Vet Microbiol 204:35–42 28532803 10.1016/j.vetmic.2017.03.035

[64] Schroder K, Tschopp J (2010) The inflammasomes. Cell 140:821–832 20303873 10.1016/j.cell.2010.01.040

[65] He Y, Hara H, Nunez G (2016) Mechanism and regulation of NLRP3 inflammasome activation. Trends Biochem Sci 41:1012–1021 27669650 10.1016/j.tibs.2016.09.002 PMC5123939

[66] Armstead WM, Bohman LE, Riley J, Yarovoi S, Higazi AA, Cines DB (2013) tPA-S(481)A prevents impairment of cerebrovascular autoregulation by endogenous tPA after traumatic brain injury by upregulating p38 MAPK and inhibiting ET-1. J Neurotrauma 30:1898–1907 23731391 10.1089/neu.2013.2962 PMC3814982

[67] Zhang A, Duan H, Li N, Zhao L, Pu F, Huang B, Wu C, Nan Y, Du T, Mu Y, Zhao Q, Sun Y, Zhang G, Hiscox JA, Zhou EM, Xiao S (2017) Heme oxygenase-1 metabolite biliverdin, not iron, inhibits porcine reproductive and respiratory syndrome virus replication. Free Radic Biol Med 102:149–161 27908781 10.1016/j.freeradbiomed.2016.11.044

