# Supplementary figures and images for: Salvianolic acid A inhibits PRRSV replication via binding to Keap1 to activate the MKRN1–Nrf2–NQO1 pathway
Source: Vet Res. 2025 Sep 25;56:182. doi: 10.1186/s13567-025-01614-9 (PMC12465779; doi:10.1186/s13567-025-01614-9)

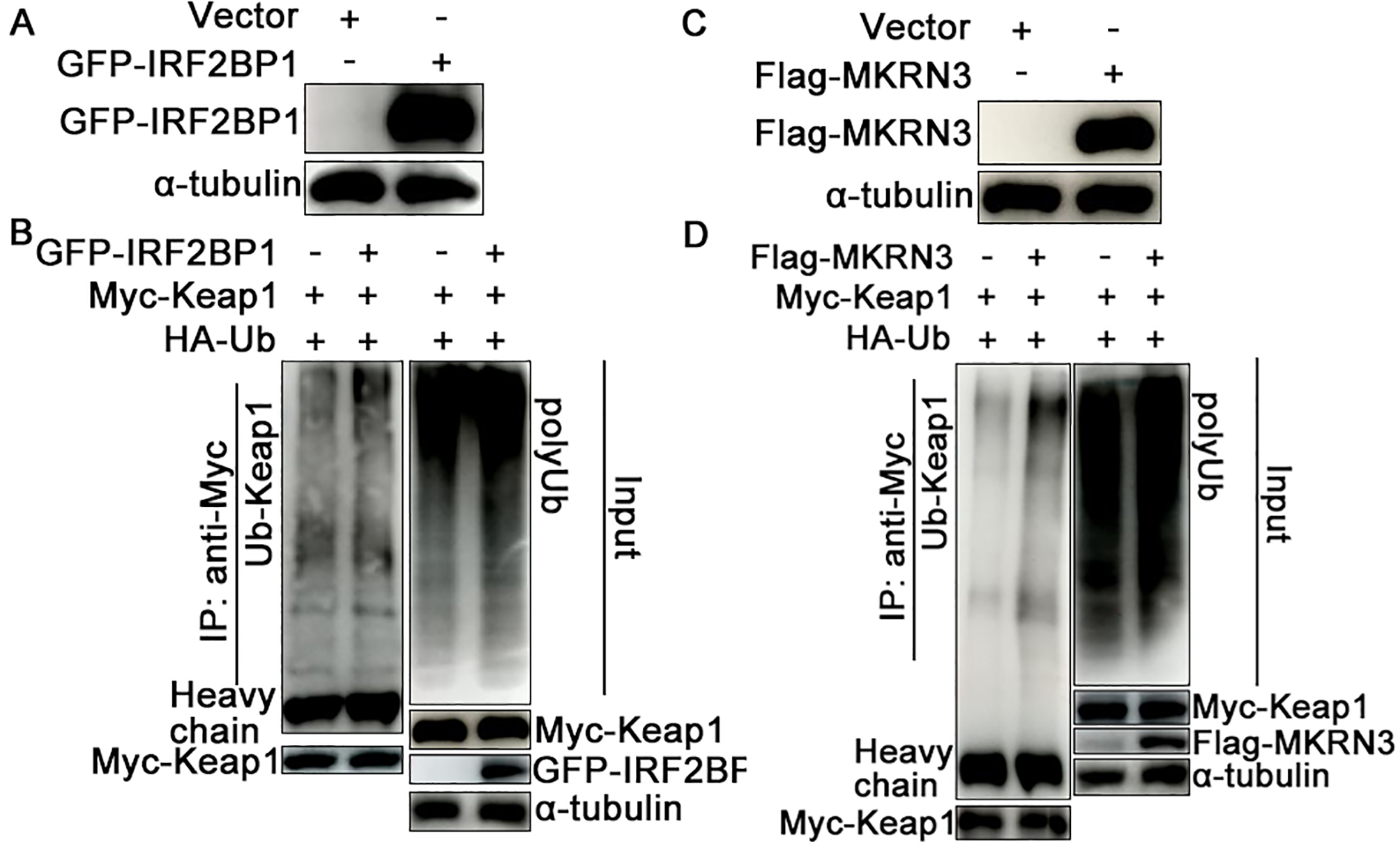

Supplement: Supplementary file 1 — Additional file 1: IRF2BP1 or MKRN3 did not catalyze the ubiquitination of Keap1. (A) Verification of the expression of recombinant Flag-MKRN3. (B) HEK293T cells were cotransfected with Flag-MKRN3 or Myc-Keap1 together with HA-Ub for 48 h, after which the cells were harvested for analysis of Keap1 ubiquitination usin a co-IP assay. (C) Verification of the expression of recombinant GFP-IRF2BP1. (D) Effect of IRF2BP1 overexpression on the ubiquitination of Keap1. [file 13567_2025_1614_MOESM1_ESM.tif]

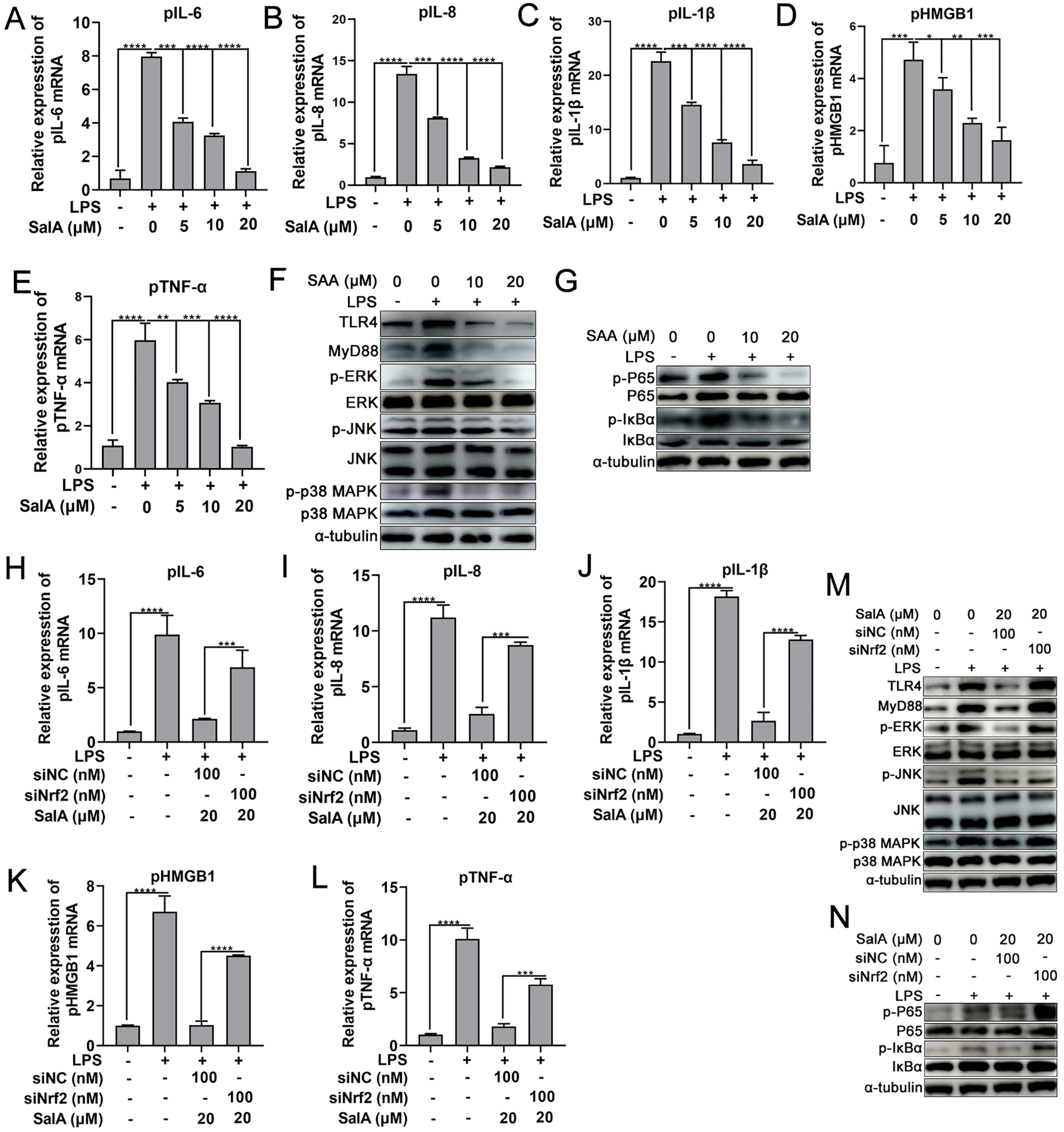

Supplement: Supplementary file 2 — Additional file 2: SalA inhibits LPS-induced inflammation- and pyroptosis-related signalling pathways by activating the transcription factor Nrf2. PAMs were treated with LPS (1 mg/mL) for 12 h and then treated with 0, 5, 10, or 20 μM SalA. After treatment for 24 h, the cells were harvested. The expression of pIL-6 (A), pIL-8 (B), pIL-1β (C), pHMGB1 (D) and pTNF-α (E) was detected by qRT-PCR. (F) Effect of SalA on LPS-induced TLR4/MyD88-MAPK pathway activation. (G) Effect of SalA on LPS-induced NF-κB pathway activation. Effect of Nrf2 knockdown on the inhibitory effects of SalA on pIL-6 (H), pIL-8 (I), pIL-1β (J), pHMGB1 (K) and pTNF-α (L) levels. (O) Effect of Nrf2 knockdown on SalA-mediated inhibition of the TLR4/MyD88-MAPK pathway. (P) Effect of Nrf2 knockdown on SalA-mediated inhibition of the NF-κB pathway. [file 13567_2025_1614_MOESM2_ESM.tif]

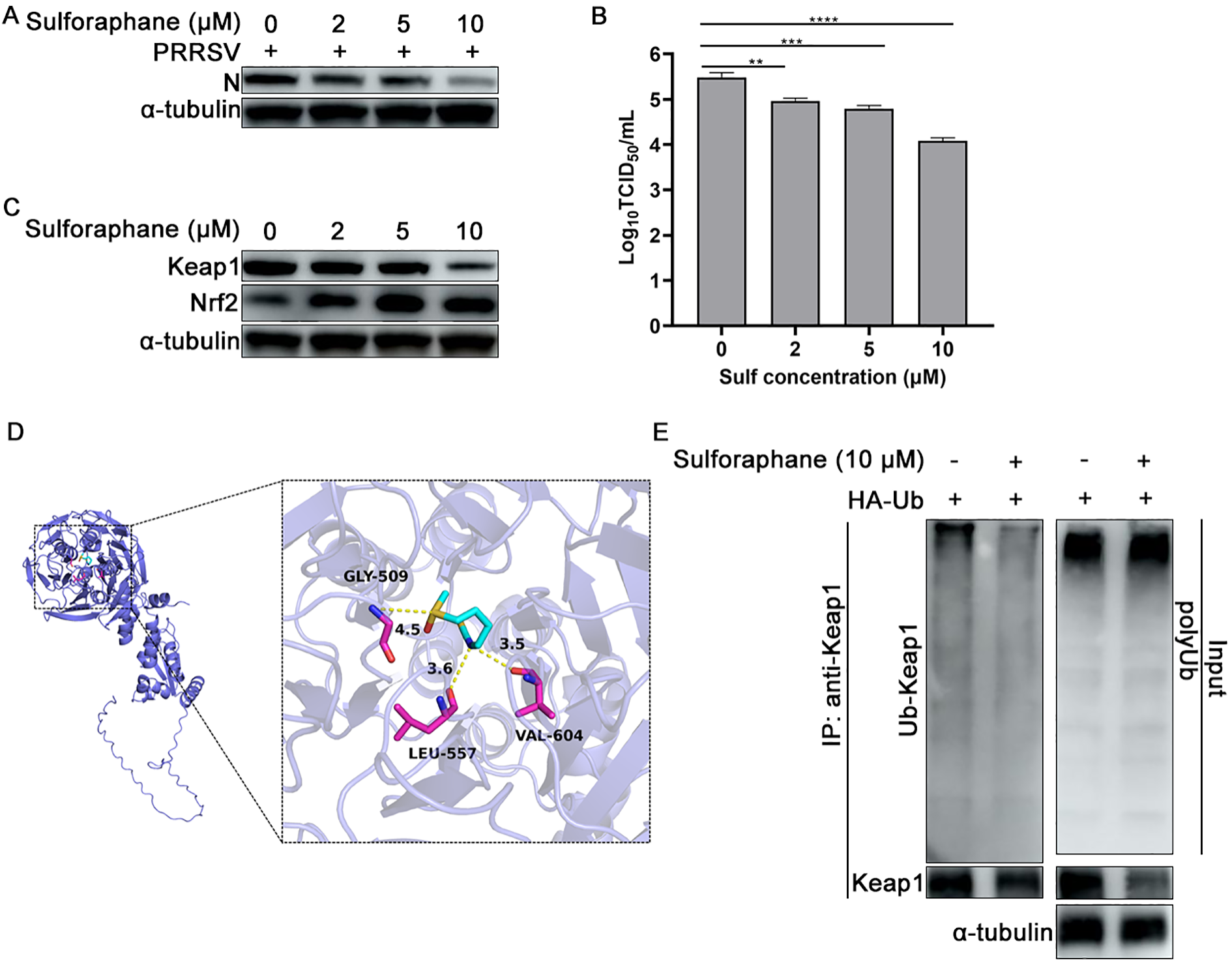

Supplement: Supplementary file 3 — Additional file 3: Effects of sulforaphane on PRRSV replication and the activity of the Keap1-Nrf2 pathway. Effect of sulforaphane on PRRSV replication in MARC-145 cells: (A) expression of the PRRSV N protein; (B) supernatant progeny viral titres. (C) Effects of sulforaphane on Keap1-Nrf2 pathway activity. (D) Docked conformation of sulforaphane with Keap1. (E) Effect of sulforaphane on the ubiquitination of Keap1. [file 13567_2025_1614_MOESM3_ESM.tif]
